# Computational Investigation of Cinnamon Phytochemicals Targeting Key Cancer Signaling Pathways: Molecular Docking, ADMET and Molecular Dynamics Simulations Analysis

**DOI:** 10.3390/cimb48080807

**Published:** 2026-08-10

**Authors:** Ravindra Raut, Shehwaz Anwar, Reem A. Alromaihi, Faris Alrumaihi

**Affiliations:** 1 Department of Medical Laboratory Technology, Mohan Institute of Nursing and Paramedical Sciences, Bareilly 243302, Indiashehwazanwar25@gmail.com (S.A.); 2 Department of Medical Laboratories, College of Applied Medical Sciences, Qassim University, Buraydah 51452, Saudi Arabia

**Keywords:** *Cinnamomum* spp., colorectal cancer, phytochemicals, consensus molecular docking, PI3K, NF-κB, mTOR, molecular dynamics simulation, MM/GBSA, ADMET, computational pharmacology

## Abstract

Cancer remains one of the leading causes of morbidity and mortality worldwide, highlighting the need for safe and effective therapeutic strategies targeting multiple oncogenic pathways. Cinnamon (*Cinnamomum* spp.) contains several bioactive phytochemicals with reported antioxidant and anticancer properties; however, their potential interactions with key cancer-associated signaling proteins have not been comprehensively investigated. In this study, an integrated computational and preliminary experimental approach was employed to evaluate four major cinnamon phytochemicals, namely e-cinnamaldehyde, eugenol, p-cymene, and cinnamic acid. Consensus molecular docking was performed using AutoDock Vina (v1.2.7), Smina (v2020.12.10), and GNINA (v1.3.3) against phosphoinositide 3-kinase (PI3K), nuclear factor kappa B (NF-κB), and mammalian target of rapamycin (mTOR). Docking analyses were complemented by protein-ligand interaction profiling, pharmacokinetic and toxicity prediction (ADMET), and a 100 ns molecular dynamics simulation with MM/GBSA binding free-energy analysis of the selected mTOR-p-cymene complex. In addition, the antioxidant activity and cytotoxic effects of a crude methanolic cinnamon bark extract were evaluated using in vitro antioxidant assays and MTT assays against HCT-116 and HT-29 colorectal cancer cell lines. Consensus docking predicted that all four phytochemicals were capable of interacting with the selected protein targets, although the predicted binding profiles varied among the compounds. Eugenol showed comparatively more favorable predicted interactions with PI3K, p-cymene produced the lowest predicted docking score for NF-κB, and cinnamic acid displayed a comparatively consistent predicted multitarget binding profile across PI3K, NF-κB, and mTOR. ADMET analysis suggested that all compounds satisfied major drug-likeness criteria and exhibited predicted oral bioavailability, although potential cytochrome P450 interactions and hepatotoxicity were predicted for some compounds. Molecular dynamics simulation indicated that the selected mTOR-p-cymene complex maintained a stable binding pose throughout the simulation, while MM/GBSA analysis yielded a modest binding free-energy estimate (ΔG_bind = −4.70 ± 8.20 kcal/mol), which should be interpreted cautiously because of the observed energetic variability. The crude methanolic cinnamon bark extract exhibited antioxidant activity and reduced the viability of HCT-116 and HT-29 colorectal cancer cells in a concentration-dependent manner. Collectively, these findings provide computational predictions of potential interactions between selected cinnamon-derived phytochemicals and cancer-associated signaling proteins and are consistent with the preliminary observation that the crude cinnamon extract exhibits antioxidant activity and cytotoxic effects in colorectal cancer cell lines. However, the computational analyses do not establish direct inhibition of the PI3K/NF-κB/mTOR signaling pathway, and the biological assays were performed using a crude extract rather than isolated phytochemicals. Therefore, further studies using purified compounds, biochemical target validation, pathway-specific cellular analyses, and in vivo models are required to determine whether the predicted protein-ligand interactions contribute to the observed biological activity.

## 1. Introduction

Natural products derived from medicinal plants have long been recognized as valuable sources of therapeutic agents for the prevention and treatment of various diseases, including cancer. In recent years, interest in plant-derived bioactive compounds has increased because of their structural diversity, potentially favorable safety profiles, and ability to modulate multiple molecular pathways involved in the progression of various diseases. For example, apigenin has been reported to induce apoptosis and autophagy by inhibiting the PI3K/AKT/mTOR pathway, modulating NF-κB activation, and suppressing Wnt/β-catenin signaling [1,2].

Cinnamon (Cinnamomum spp.), a member of the Lauraceae family, is one of the most widely used spices worldwide and has been employed in traditional medicine for centuries, including Cinnamomum loureiroi, Cinnamomum burmannii, Cinnamomum cassia, and Cinnamomum verum [3]. Different parts of the cinnamon plant, particularly the bark, leaves, flowers, and fruits, are used to extract essential oils and bioactive compounds such as cinnamaldehyde, eugenol, camphor, and trans-cinnamyl acetate. These compounds have been reported to possess a wide range of biological activities, including anti-inflammatory, anti-diabetic, antioxidant, antimicrobial, and anticancer properties [4,5].

The unchecked proliferation and spread of aberrant cells that invade neighboring tissues and travel to distant organs are hallmarks of cancer and remain one of the biggest threats to global health [6,7]. External agents, such as chemotoxins and physical and biological factors, interact with an individual’s genetic factors to induce these changes. Despite significant advances in chemotherapy and targeted therapies, many anticancer drugs are associated with side effects, toxicity, and therapeutic resistance. Therefore, there is an urgent need for new strategies to increase tolerance and reduce the side effects of chemotherapy drugs. This research topic focuses on this problem and identifies several advancements in the field [8].

Consequently, extensive research has been conducted on the use of herbal remedies, either alone or in conjunction with chemotherapy, for cancer treatment [9]. However, for the development of cinnamon as a traditional medicine for cancer treatment, further studies are necessary, such as elucidating the working mechanisms and characterizing the active compounds directly linked to anti-tumor activity [9]. Recent studies have demonstrated that cinnamon extracts and their phytochemical constituents exhibit potential anticancer activities through multiple mechanisms, including the inhibition of tumor cell proliferation, induction of apoptosis, and suppression of angiogenesis [10].

Cinnamon extract and its derived compounds have been reported to modulate important molecular signaling pathways involved in cancer progression, such as the nuclear factor-κB (NF-κB) and phosphatidylinositol-3 kinase (PI3K/AKT/mTOR) signaling pathways [11,12,13]. Dysregulation of these pathways plays a crucial role in tumor development by promoting cell survival, inflammation, angiogenesis, and resistance to apoptosis [14,15]. PI3K, NF-κB, and mTOR were selected because they constitute three major, functionally interconnected signaling hubs that regulate multiple hallmarks of cancer, including uncontrolled proliferation, apoptosis evasion, inflammation, metabolic reprogramming, angiogenesis, and therapeutic resistance [16]. Activation of PI3K stimulates the downstream AKT/mTOR signaling cascade, whereas extensive crosstalk between PI3K/AKT/mTOR and NF-κB coordinates tumor cell survival and inflammatory responses [17,18,19,20]. Owing to this close functional relationship, simultaneous modulation of these pathways has emerged as an attractive strategy for the development of multitarget anticancer therapies. Although other oncogenic regulators, such as EGFR, KRAS, G12R, BRAF, MEK, VEGFR, AKT, and β-catenin, also play important roles in cancer progression [21,22,23,24,25,26], the present study focused on representative signaling hubs within the PI3K/NF-κB/mTOR network to establish a proof-of-concept framework for evaluating cinnamon-derived phytochemicals as potential multitarget anticancer agents.

Among the bioactive constituents of cinnamon, e-cinnamaldehyde, eugenol, p-cymene, and cinnamic acid have attracted considerable attention because of their diverse pharmacological activities. It has been demonstrated that aqueous cinnamon extract possesses anti-angiogenic qualities and downregulates pro-angiogenic factors. Inhibition of cyclooxygenase (COX-2) both in vitro and in vivo, as well as hypoxia-inducible factor 1-alpha (HIF-1α), signal transducer and activator of transcription-3 (STAT3), and AKT protein kinase B (PBK), affects the expression of vascular endothelial growth factor (VEGF) during different stages of angiogenesis [27].

Recently, computer-aided drug design (CADD) has emerged as a powerful strategy for identifying potential therapeutic compounds and predicting their interactions with biological targets [28]. CADD techniques can reduce the time and expense of drug discovery and development and help researchers understand the molecular principles of therapeutic action and drug toxicity. Molecular docking [29] is a method for predicting a ligand’s binding mechanism and affinity for a target protein. It is a powerful computational technique in structural biology and computer-aided drug design that predicts the affinities and modes of binding of small drug-like molecules to biological targets during drug development [30,31]. It predicts a molecule’s binding prototype to a receptor and is one of the most important aspects of modern drug discovery [32,33].

Therefore, this study employed an integrated in silico approach to evaluate the anticancer potential of selected cinnamon-derived phytochemicals. The interactions of cinnamaldehyde, eugenol, p-cymene, and cinnamic acid with key cancer-related targets (PI3K, NF-κB, and mTOR) were investigated using molecular docking analyses. The stability and dynamic behavior of the protein-ligand complexes were further assessed using molecular dynamics simulations. In addition, pharmacokinetic and toxicity properties were evaluated using ADMET analysis to determine drug likeness and safety profile. To support the computational findings, the methanolic extract of cinnamon was further evaluated for its cytotoxic and anti-proliferative effects against HCT-116 and HT29 colorectal cancer cell lines.

## 2. Materials and Methods

### 2.1. Docking Investigations

A standardized and widely accepted computational workflow was employed to ensure the reproducibility and reliability of the screening process. These methods were selected to provide consistent and comparative evaluations of ligand-protein interactions across several targets.

To investigate the potential interactions between cinnamon-derived phytochemicals and cancer-related targets, three signaling proteins were selected: PI3K, NF-κB, and mTOR, which are involved in tumor progression. The three-dimensional structures of the target proteins were obtained from the Protein Data Bank (https://www.rcsb.org/, accessed on 15 January 2026).

The selection of protein structures was based on crystallographic resolution, source organism, availability of ligand-bound information for active site identification, and presence of key functional residues in the active site. The protein structures were downloaded in PDB format and further processed for docking studies. The active biomolecules reported in cinnamon, including e-cinnamaldehyde, eugenol, p-cymene, and cinnamic acid, were selected as ligands [34,35]. The three-dimensional structures of these ligands were obtained from the NCBI PubChem compound database (https://pubchem.ncbi.nlm.nih.gov/, accessed on 15 January 2026), which provided the structures of all ligands in the Structure Data File (SDF) format.

#### 2.1.1. Protein Retrieval and Preparation

The protein structures were carefully selected to ensure reliable molecular docking analyses. The selection criteria included the availability of experimentally determined X-ray crystal structures, the presence of co-crystallized ligands to facilitate accurate definition of the binding site, structural completeness without missing key active-site residues, and the biological relevance of the protein conformation. Based on these criteria, PI3K (PDB ID: 1E7U), NF-κB (PDB ID: 3RZF), and mTOR (PDB ID: 4JT6) were selected. These structures were determined by X-ray diffraction with crystallographic resolutions of 2.00 Å, 4.00 Å, and 3.60 Å, respectively [36,37,38]. Although the NF-κB and mTOR structures have moderate crystallographic resolutions, both contain co-crystallized ligands and well-defined binding pockets, making them suitable for structure-based molecular docking studies.

Protein preparations were performed to ensure structural integrity and compatibility with the molecular docking simulations. Protein structures were obtained from the Protein Data Bank (PDB) using the ‘rcsbsearchapi’ Python library combined with the ‘requests’ module for API access and ‘os’ for handling the file paths and directories. Searches were performed based on the Enzyme Commission (EC) numbers (https://www.brenda-enzymes.org/all_enzymes.php, accessed on 15 January 2026) and ligand-specific queries, respectively.

The downloaded structures were parsed and analyzed using the Python library MDAnalysis (v2.10.0) (https://www.mdanalysis.org/, accessed on 15 January 2026) [39], which facilitated atom selection, structural inspection, and the manipulation of files. Missing atoms and hydrogens were added, and the protein structure was optimized at physiological pH using the command-line tool pdb2pqr (v3.7.1) (https://pdb2pqr.readthedocs.io/en/latest/, accessed on 15 January 2026) [40]. Visualization and preliminary structural validation during preprocessing were performed using the nglview tool (v4.0) (https://github.com/nglviewer/nglview, accessed on 15 January 2026) [41].

#### 2.1.2. Ligand Preparation

Ligand preparation was performed using the Python package Meeko (v0.7.1) (https://pypi.org/project/meeko/, accessed on 15 January 2026), a Python interface for AutoDock tools that automates ligand protonation, torsional flexibility assignment, and file format conversion required for docking [42]. Ligand structures were retrieved using requests and subsequently checked for structural integrity. This preparation step ensured proper input formatting and compatibility with AutoDock Vina (v1.2.7) (https://github.com/ccsb-scripps/AutoDock-Vina, accessed on 15 January 2026), [43] and enabled downstream visualization.

#### 2.1.3. Docking Grid Definition

The docking grid (ligand box) was defined using the Python library MDAnalysis, which enabled atom-based selection and identification of active site-binding pockets. The grid center coordinates and box dimensions were selected based on the positions of the co-crystallized ligands and key active site residues to ensure complete coverage of the catalytic pockets while minimizing the excess search space. Docking box validation was further assessed using native ligand redocking with the identical docking parameters.

#### 2.1.4. Molecular Docking

Molecular docking simulations were conducted using ‘vina’, a Python module within the AutoDock-Vina (v1.2.7) package (https://github.com/ccsb-scripps/AutoDock-Vina, accessed on 15 January 2026), with an exhaustiveness parameter set to 32 to enhance sampling, resulting in the generation of up to five poses for each ligand. The docking runs produced binding energies and conformations that were systematically recorded. Docking scores were used for relative comparison among the tested phytocompounds rather than direct comparison with the indicated inhibitors. As docking energies provide only approximate estimates of binding affinity, the results were interpreted cautiously within a preliminary screening framework.

#### 2.1.5. Post-Docking Analysis and Visualization

The docking results, including the predicted binding affinities, were stored and processed using Pandas [44] for tabular data management. The docked ligand conformations were analyzed using RDKit (v2025.09.5) (https://www.rdkit.org/, accessed on 15 January 2026) and Prolif (v2.2.1) (https://prolif.readthedocs.io/en/stable/, accessed on 15 January 2026) [45], which generates an interaction fingerprint for the interactions between a protein and different docking poses. Structural and interaction visualizations were performed using MDAnalysis and nglview, facilitating the qualitative inspection and quantitative comparison of the binding modes.

The docking conformation of each ligand was determined based on the pose with the highest binding affinity, corresponding to the most negative Gibbs free energy of binding (ΔG). Among the multiple docking poses generated, only those with the lowest ΔG values were selected for analysis. These best-scoring conformations, represented by negative free energy values, were considered to reflect the most favorable and stable ligand-protein interactions.

#### 2.1.6. Workflow Integration

The workflow was implemented in a Jupyter Notebook (v7.6.1) (https://jupyter.org/, accessed on 15 January 2026) [46] by integrating Python libraries and the Anaconda environment (v26.1.1) (https://www.anaconda.com/, accessed on 15 January 2026). This setup provided a reproducible environment for protein retrieval, structural preparation, docking simulation, and post-docking analysis. The combination of programmatic control, reproducibility, and visualization capabilities ensured the efficiency and transparency of the docking studies.

The molecular docking protocol was supported by redocking the native co-crystallized ligands into the active sites of PI3K (1E7U), NF-κB (3RZF), and mTOR (4JT6) using the same docking parameters employed for the cinnamon-derived phytocompounds. The predicted binding poses were compared with the crystallographic ligand conformations, and both the best-pose RMSD and the average RMSD across all generated docking poses were recorded. Best-pose RMSD values below 2.0 Å were considered indicative of successful reproduction of the experimental binding geometry.

#### 2.1.7. Consensus Molecular Docking

Initial molecular docking was performed using AutoDock Vina (v1.2.7) to estimate binding affinities. The docked complexes were subsequently refined using Smina (v2020.12.10) (https://github.com/mwojcikowski/smina, accessed on 24 June 2026) [47], an enhanced implementation of the AutoDock Vina scoring function. Docking calculations employed the native ligand to automatically define the search box using the --autobox_ligand option with an additional 6 Å margin (--autobox_add 6). The docking exhaustiveness was set to 32, a fixed random seed of 42 was used to ensure reproducibility, and a maximum of 20 binding poses was generated for each ligand-protein complex. The pose with the lowest predicted binding free energy was selected for subsequent analyses.

#### 2.1.8. Deep Learning-Based Rescoring

To improve docking reliability, the best docked complexes obtained from Smina were independently rescored using GNINA (v1.3.3) (https://github.com/gnina/gnina, accessed on 26 June 2026) [48], which integrates convolutional neural networks with classical molecular docking. GNINA generated refined binding affinities together with CNN pose scores and CNN-predicted binding affinities. The CNN pose score ranges from 0 to 1, with higher values indicating greater confidence in the predicted ligand orientation within the binding pocket. For each ligand-protein pair, the top-ranked pose generated by GNINA was retained for downstream analyses.

#### 2.1.9. Consensus Docking Analysis

A consensus docking strategy was employed by integrating the results obtained from AutoDock Vina, Smina, and GNINA. Classical docking scores (AutoDock Vina and Smina), GNINA-refined binding affinity, CNN pose score, and CNN-predicted affinity were collectively considered to evaluate ligand binding performance. Rather than relying on a single scoring function, ligand ranking was based on the combined agreement among these complementary scoring metrics, thereby reducing potential bias associated with individual docking algorithms and improving confidence in the predicted protein-ligand interactions. All docking calculations were performed using identical docking parameters for every ligand-protein complex to ensure methodological consistency and facilitate direct comparison of binding affinities across targets.

### 2.2. Pharmacokinetics Investigations

#### ADMET Analysis

The pharmacokinetic and toxicity profiles of the selected ligand molecules were evaluated using in silico Absorption, Distribution, Metabolism, Excretion, and Toxicity (ADMET) analyses to predict their drug-likeness and safety characteristics. The analysis was conducted using freely available online platforms, including SwissADME (https://www.swissadme.ch/, accessed on 25 January 2026), ADMETlab3.0 (https://admetlab3.scbdd.com/, accessed on 25 January 2026), and PreADMET (https://preadmet.webservice.bmdrc.org/, accessed on 25 January 2026).

The 3D structures (MOL format) of the ligands were retrieved from MolView (https://molview.org/, accessed on 25 January 2026) and converted to SMILES notation for ADMET analysis. The SwissADME server was used to estimate fundamental pharmacokinetic parameters, such as molecular weight, hydrogen bond donors and acceptors, topological polar surface area (TPSA), lipophilicity (logP), solubility (logS), and compliance with Lipinski’s rule of five. The BOILED-Egg model predicts gastrointestinal absorption and blood–brain barrier (BBB) permeability.

ADMETlab 3.0 and PreADMET were used to predict advanced pharmacokinetic descriptors, including plasma protein binding (PPB), P-glycoprotein (P-gp) substrate/inhibitor potential, cytochrome P450 (CYP450) enzyme interactions, half-life (t_1/2_), and clearance rates. Toxicity parameters, including Ames mutagenicity, carcinogenicity, and hepatotoxicity, were assessed to evaluate the safety profiles of the compounds.

All obtained data were compiled and analyzed to determine whether the ligands met acceptable pharmacokinetic thresholds, thereby identifying potential lead molecules with favorable absorption, distribution, metabolism, and excretion characteristics and minimal toxicity risks.

### 2.3. Molecular Dynamics Simulation

Molecular dynamics (MD) simulation was performed to investigate the structural stability, conformational dynamics, and binding behavior of the selected mTOR–p-cymene protein–ligand complex. All simulations were carried out using GROMACS (v2025.1) (https://www.gromacs.org/, accessed on 15 May 2026) [49] with the CHARMM36 force field for the protein and SwissParam-generated parameters for the ligand. The protein was parameterized using the CHARMM36 force field, and ligand topologies were generated using SwissParam (https://swissparam.ch/, accessed on 15 May 2026) [50]. Prior to the simulation, the systems were subjected to energy minimization using the steepest descent algorithm for 2500 steps to remove steric clashes and optimize the geometry. Each system was solvated using the SPC water model in a cubic box with periodic boundary conditions (PBC). Counter ions (Na^+^ and Cl^−^) were added using gmx genion to ensure overall charge neutrality.

After minimization, a two-stage equilibration was performed. First, a 100 ps NVT equilibration was carried out using the v-rescale thermostat to stabilize the temperature at 310 K. This was followed by a 100 ps NPT equilibration using the Parrinello–Rahman barostat to maintain pressure at 1 bar and achieve density equilibration [51]. Position restraints were applied to the heavy atoms of the protein during equilibration. Bond constraints were imposed using the LINCS algorithm, and long-range electrostatic interactions were treated using the Particle Mesh Ewald (PME) method [52]. Following equilibration, a single unrestrained 100 ns production molecular dynamics simulation was performed for the selected mTOR–p-cymene protein–ligand complex. Trajectory coordinates were saved at regular intervals for subsequent analyses, including root mean square deviation (RMSD), root mean square fluctuation (RMSF), radius of gyration (Rg), solvent-accessible surface area (SASA), hydrogen-bond analysis, and MM/GBSA binding free-energy calculations.

#### 2.3.1. Root Mean Square Deviation (RMSD)

The RMSD is a metric that quantifies the distance between frames. This is determined for each profile frame. The root mean square deviation of frame x was calculated.

$${RMSD}_{X}=\sqrt{\frac{1}{N}\sum_{i=1}^{N}{\left(r_{i}^{’}\left(t_{x}\right)\right)-r_{i}\left(t_{ref}\right)}^{2}}$$

where N is the number of selected atoms, r_i_ (t_ref_) represents the position of atom I in the reference structures, and denotes the position of the same atom in frame x after optimal structural superposition onto the reference. All RMSD calculations were performed using the gmx function in GROMACS (v2025.1), and the resulting trajectories were visualized using XmGrace (v5.1.25) (https://plasma-gate.weizmann.ac.il/Grace/, accessed on 20 May 2026) [53].

#### 2.3.2. Root Mean Square Fluctuation (RMSF)

RMSF values can be used to determine the local flexibility based on residue displacements during the MD simulation. The Root Mean Square Fluctuation (RMSF) is useful for characterizing local changes along the protein chain. The RMSF for residue i was calculated as follows:

$${RMSF}_{I}=\sqrt{\frac{1}{T}\sum_{I=1}^{T}{\left(r_{i}\left(t\right)-(r_{i})\right)}^{2}}$$

where $r_{i}$(t) represents the position I at time t and r_i_ is its time-averaged position. Unlike the RMSD, which compares each frame to a reference structure, the RMSF characterizes local fluctuations around the mean trajectory. Peaks in the RMSF profile correspond to flexible regions, typically loops and the N- or C-terminal segments, whereas lower values indicate more rigid, structurally stable regions of the protein [54,55].

#### 2.3.3. Radius of Gyration (Rg)

The radius of gyration (Rg) represents the mass-weighted root-mean-square distance of a collection of atoms from their common center of mass. Rg is widely used to evaluate the global compactness and folding stability of biomolecular systems during MD simulations. A stable Rg profile indicates maintenance of the tertiary structure, whereas significant deviations imply expansion, unfolding, or structural reorganization. In this study, Rg was calculated for each trajectory frame using GROMACS analysis tools to monitor the compactness of the protein and protein–ligand complexes over a 100 ns simulation [56].

#### 2.3.4. Solvent Accessible Surface Area (SASA)

The Solvent Accessible Surface Area (SASA) was computed using the gmx sasa module in GROMACS (v2025.1) along the 100 ns MD trajectory. SASA provides insights into the extent of solvent exposure and potential structural rearrangements at the protein surface. A default probe radius of 0.14 nm was applied, corresponding to the radius of a water molecule. SASA values were extracted at regular intervals for the entire protein–ligand complex to evaluate dynamic changes in surface exposure, folding stability, and solvent interactions throughout the simulation.

#### 2.3.5. Hydrogen Bond

The hydrogen-bond network during the 100 ns molecular dynamics simulation was analyzed using the gmx hbond module implemented in GROMACS (v2025.1). Hydrogen bonds were identified using the default geometric criteria of a donor–acceptor distance ≤ 0.35 nm and a donor–hydrogen–acceptor angle ≥ 120°. The total number of hydrogen bonds was calculated for each trajectory frame to monitor the stability and evolution of the hydrogen-bonding network throughout the simulation. Because p-cymene is an aromatic hydrocarbon lacking hydrogen-bond donor and acceptor atoms, the hydrogen-bond analysis was not interpreted as representing direct protein–ligand hydrogen bonds. Instead, the analysis was used to assess changes in the overall hydrogen-bonding environment of the simulated protein–ligand system during the molecular dynamics simulation.

#### 2.3.6. MM/GBSA Binding Free Energy

To estimate the binding free energy between the ligand and the protein, Molecular Mechanics/Generalized Born Surface Area (MM/GBSA) calculations were performed using the gmx_MMPBSA (v1.6.3) (https://github.com/Valdes-Tresanco-MS/gmx_MMPBSA, accessed on 22 May 2026) which interfaces GROMACS trajectories with AMBER free-energy calculations. This method decomposes the binding free energy (ΔG_bind) into molecular mechanical energy terms (van der Waals and electrostatic interactions) and solvation free-energy contributions (polar and nonpolar), according to the following equation:

$$∆G_{bind}=∆E_{vdW}+∆E_{ele}+∆G_{polar}+∆G_{nonpolar}$$

The Generalized Born (GB) implicit solvent model was used to compute the polar solvation energy, with the igb = 5 parameter corresponding to the GB-Neck2 model. The nonpolar solvation term (ΔG_nonpolar) was estimated from the solvent-accessible surface area using the LCPO method. Entropic contributions were omitted, which is common practice for comparative binding affinity studies and does not affect the relative ranking of ligands [40].

A total of 1001 snapshots were extracted uniformly from the 100 ns production trajectory. MM/GBSA calculations were carried out separately for the complex, the receptor (protein only), and the ligand. The binding free energy was calculated using the standard thermodynamic cycle:

$$∆G_{bind}=G_{complex}-(G_{receptor}+G_{ligand})$$

This protocol enabled detailed decomposition of binding contributions, including van der Waals, electrostatic, polar, and nonpolar solvation components, providing a robust energetic evaluation of ligand–protein interactions.

### 2.4. Preliminary In Vitro Evaluation of Cinnamon Extracts

To complement the computational findings and provide initial experimental support for the biological activity of cinnamon-derived phytocompounds, preliminary in vitro antioxidant and anti-inflammatory assays were performed using methanolic cinnamon bark extracts. As oxidative stress and inflammation play important roles in cancer progression, these assays were included to preliminarily evaluate the potential therapeutic relevance of cinnamon extracts.

#### 2.4.1. Materials

Trichloroacetic acid, ascorbic acid, 2,2-diphenyl-1-picrylhydrazyl (DPPH), ferric chloride, Folin–Ciocalteu Reagent (FCR), potassium ferricyanide, gallic acid, quercetin, and trypsin were purchased from Sigma Co., St. Louis, MO, USA. Hydrochloric acid, aluminum chloride, monosodium dihydrogen phosphate, DMSO, ethanol, methanol, disodium hydrogen phosphate, sodium hydroxide, sodium carbonate, and hydrogen peroxide were purchased from Merck, Darmstadt, Germany. All the reagents and chemicals were of analytical grade. The solvents used were of HPLC grade.

#### 2.4.2. Cinnamon Bark Extract Preparation

Cinnamon bark was bought from the local market in Bareilly, India. Cinnamon bark was first cleaned and chopped into small pieces. A drying cabinet was used to dry the cinnamon bark for three to four days at 40 to 42 °C. The plant material was dried and then processed into a powder, and 100 g of crude cinnamon bark powder was kept in a flask. The flask was filled with 1000 mL of 97% methanol solvent in a magnetic shaker for 3 h at 37 °C. The extracts were purified by filtration before being condensed using rotary evaporators at decreased pressure and 40 °C to obtain a crude active component. The sections were stored at 4 °C for future analysis.

#### 2.4.3. Hydrogen Peroxide Reducing Activity

A 40 mM hydrogen peroxide solution (pH 7.4) was prepared using phosphate buffer. Various concentrations of the plant extract (50–600 µg/mL) and ascorbic acid as the standard (100 and 200 µg/mL) were separately mixed with 1 mL of the hydrogen peroxide solution and incubated for 10 min. After incubation, the absorbance was measured at 230 nm using a spectrophotometer, with phosphate buffer without hydrogen peroxide serving as the blank. The hydrogen peroxide scavenging ability of the extract was then evaluated and compared with that of ascorbic acid [57].

Reducing capacity (%) = [(C − S)/C] × 100

where S represents the absorbance of the sample containing the extract and hydrogen peroxide solution, while C represents the absorbance of the control containing hydrogen peroxide solution alone.

#### 2.4.4. DPPH Assay

The free radical scavenging activity of the extract was further determined using the DPPH assay [41]. In this method, 1 mL of freshly prepared 0.3 mM DPPH solution in methanol was added to 1 mL of extract at various concentrations (50–600 μg/mL). Ascorbic acid (100 and 200 μg/mL) was used as the reference standard. The reaction mixtures were gently mixed and kept in the dark for 60 min to allow the reaction to occur. Following incubation, the absorbance was recorded at 517 nm using a spectrophotometer. A solution containing methanol instead of the extract served as the blank.

Percentage DPPH scavenging potential (%) = [(C − S)/C] × 100

where S represents the absorbance of the sample containing the extract and DPPH solution, while C represents the absorbance of the control containing DPPH solution in methanol alone.

#### 2.4.5. Heat-Induced Albumin Denaturation Inhibition

The anti-inflammatory activity of cinnamon bark extract was assessed using the albumin denaturation inhibition assay [22]. Different concentrations of the extract (50–600 µg/mL) or ibuprofen (100 and 200 µg/mL) were mixed with 1% bovine serum albumin solution, followed by incubation at 37 °C and heating at 71 °C. After cooling, absorbance was measured at 660 nm. A phosphate buffer with BSA served as the negative control, and all tests were performed in triplicate.

Heat-induced BSA denaturation inhibition (%) = [(C − S)/C] × 100

where C represents the absorbance of the control solution without extract or ibuprofen, and S represents the absorbance of the test solution containing the extract or ibuprofen.

### 2.5. Effect of Cinnamon Extract on Cell Viability and Proliferation

#### 2.5.1. Cell Culture and Treatments

The HCT-116 and HT29 human colorectal cancer cell lines were cultured in standard medium supplemented with fetal bovine serum and antibiotics at 37 °C and 5% CO_2_. Cells (5 × 10^3^) were seeded in culture plates and allowed to attach overnight, then treated with varying concentrations of methanolic cinnamon extract (0–100 µg/mL) for 24, 48, and 72 h.

#### 2.5.2. MTT Assay

Colon cancer cells were seeded into a 96-well cell culture plate at 3000–5000 cells per well, as previously reported [58], with an appropriate volume of cell culture medium added to each well. The plate was incubated overnight in a 5% CO_2_ incubator at 37 °C to allow the cells to adhere to the surface. Following the treatment period (24 h, 48 h, and 72 h) with methanolic extract of cinnamon (2.5 µg/mL to 100 µg/mL), 20 µL of 3-(4,5-dimethylthiazol-2-yl)-2,5-diphenyltetrazolium bromide (MTT) solution was added to each well, and the plate was incubated again in the 5% CO_2_ incubator at 37 °C for 2–3 h to allow the formation of formazan crystals. After incubation, the medium containing the MTT solution was carefully aspirated without disturbing the formazan crystals. To solubilize the crystals, 100 µL of dimethyl sulfoxide (DMSO) was added to each well, and the plate was gently shaken to ensure complete dissolution. The absorbance of the resulting solution was measured at 540 nm using a microplate reader to evaluate cell viability. The Percentage Cell viability was calculated using the following formula [59]:

Percentage Viability (%) = Abs. of Sample/Abs. of Control × 100

## 3. Results

### 3.1. Molecular Docking Study

**Figure 1:**
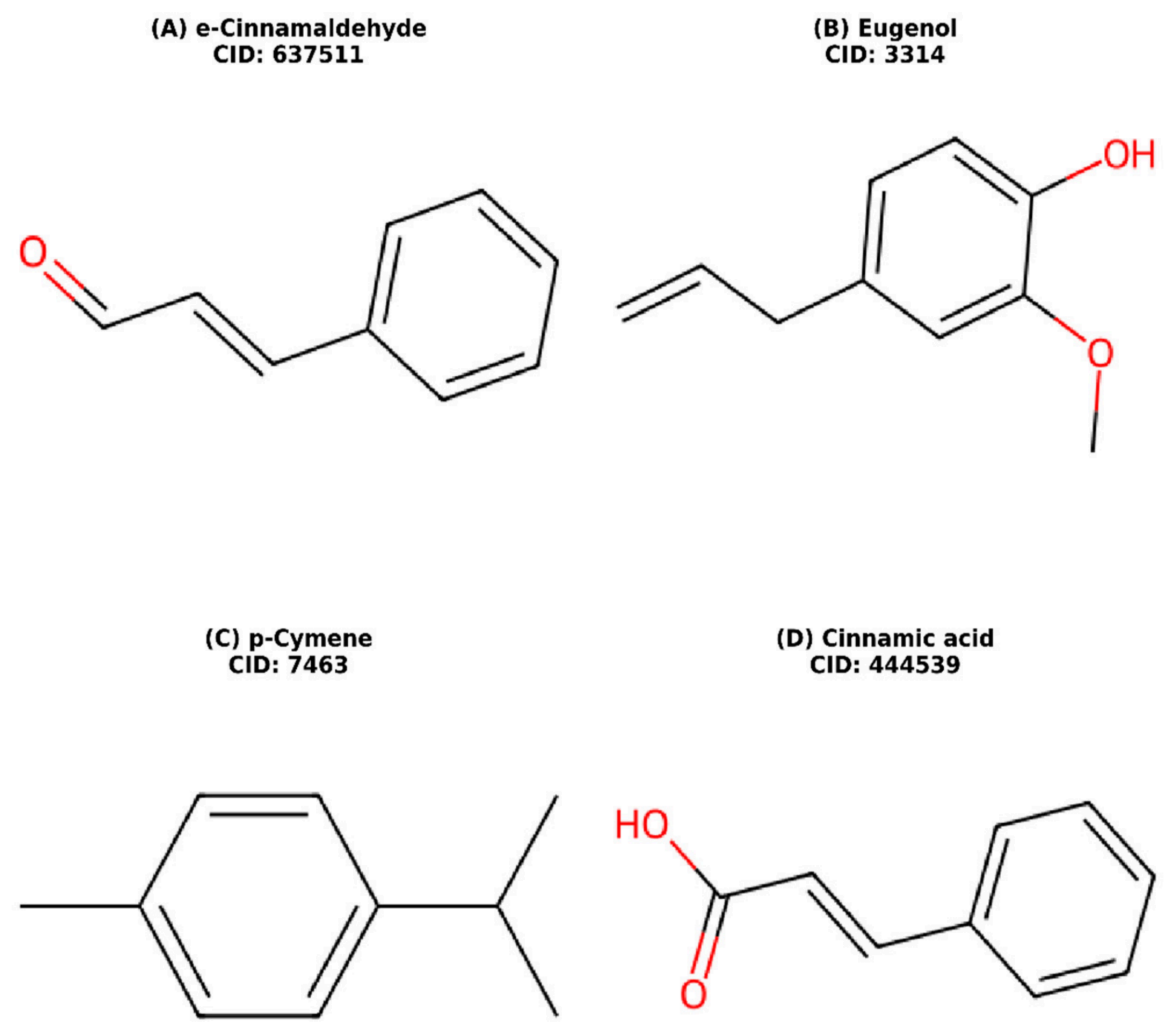
The 2D structures of selected cinnamon ligands with PubChem CID: (**A**) e-Cinnamaldehyde (637511); (**B**) Eugenol (3314); (**C**) p-Cymene (7463); and (**D**) Cinnamic acid (444539).

**Figure 2:**
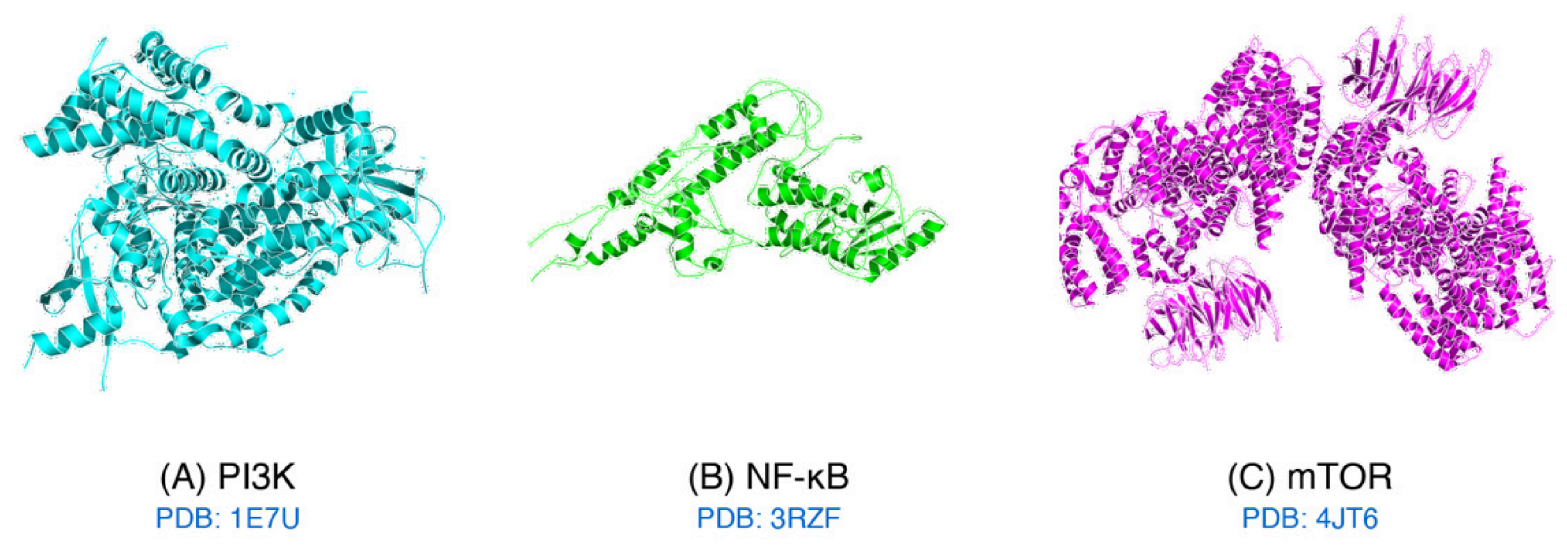
The 3D structures of selected proteins with PDB IDs: (**A**) phosphatidylinositol-3-kinase (PI3K) [1E7U]; (**B**) NF-κB [3RZF]; and (**C**) mammalian target of rapamycin (mTOR) [4JT6].

**Figure 3:**
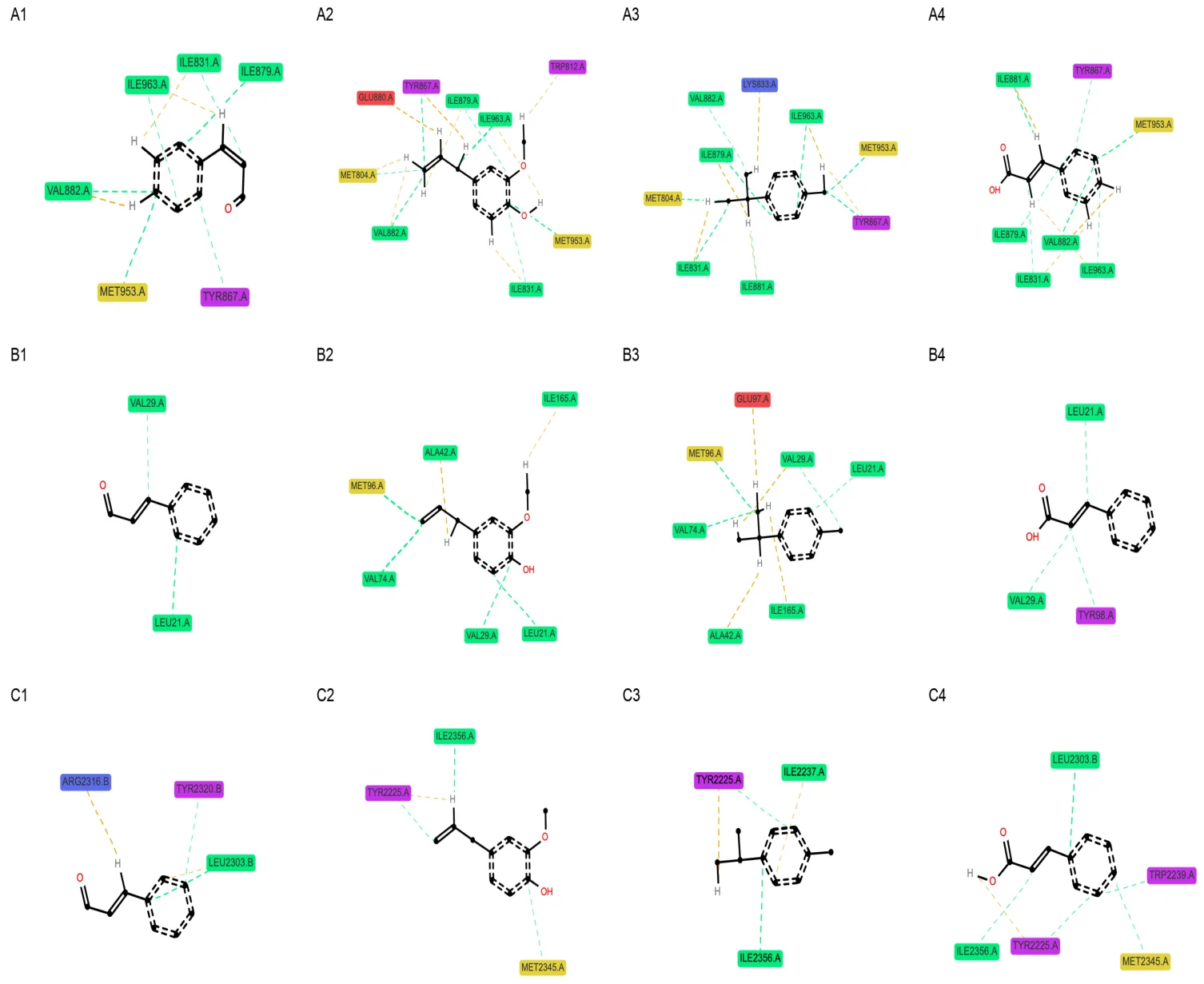
Two-dimensional interaction profiles of cinnamon-derived phytochemicals with target proteins. (**A**) Phosphatidylinositol-3-kinase (PI3K); (**B**) Nuclear factor-kappa B kinase (NF-κB); and (**C**) Mammalian target of rapamycin (mTOR). For each protein, panels (1–4) correspond to interactions with e-cinnamaldehyde, eugenol, p-cymene, and cinnamic acid, respectively (**A1**–**A4**,**B1**–**B4**,**C1**–**C4**). Within each panel, amino acid residues are highlighted with color-coded boxes: acidic residues (red), aliphatic residues (green), aromatic residues (purple), and sulfur-containing residues (yellow). Intermolecular interactions between the ligand and the target residues are indicated by colored lines: hydrophobic interactions (light green dashed lines) and van der Waals contacts (orange dashed lines).

The present study examined the molecular interactions between protein molecules, such as phosphatidylinositol-3-kinase (PI3K), NF-κB, and mammalian target of rapamycin (mTOR) (Figure 1), and the active constituents of cinnamon, including e-cinnamaldehyde, eugenol, p-cymene, and cinnamic acid (Figure 2). Table 1 displays the study details, and Figure 3 shows the molecular interactions between the ligands and proteins.

Molecular docking analysis predicted that all selected phytocompounds could interact with PI3K, NF-κB, and mTOR, with differences observed in their predicted binding energies and interaction profiles. Among the compounds evaluated, p-cymene displayed comparatively lower predicted docking energies across the three targets. Interaction analysis indicated that ligand binding to PI3K was primarily mediated by hydrophobic contacts within the catalytic pocket, suggesting that the phytocompounds occupied the predicted binding site (Table 1).

Similarly, binding to NF-κB was stabilized through interactions within the active site pocket, indicating effective ligand positioning. For mTOR, ligand binding was also stabilized within the catalytic pocket through interactions with key residues, suggesting stable ligand–protein complex formation (Table 1). Docking grids for all targets were defined around their respective active sites to ensure appropriate sampling of ligand conformations (Table 2).

These results highlight the ability of cinnamaldehyde, eugenol, p-cymene, and cinnamic acid to establish hydrogen bonding and hydrophobic contacts with catalytically relevant amino acids, with p-cymene and cinnamic acid exhibiting particularly stable interactions across all three protein targets (Figure 3).

**Figure 4:**
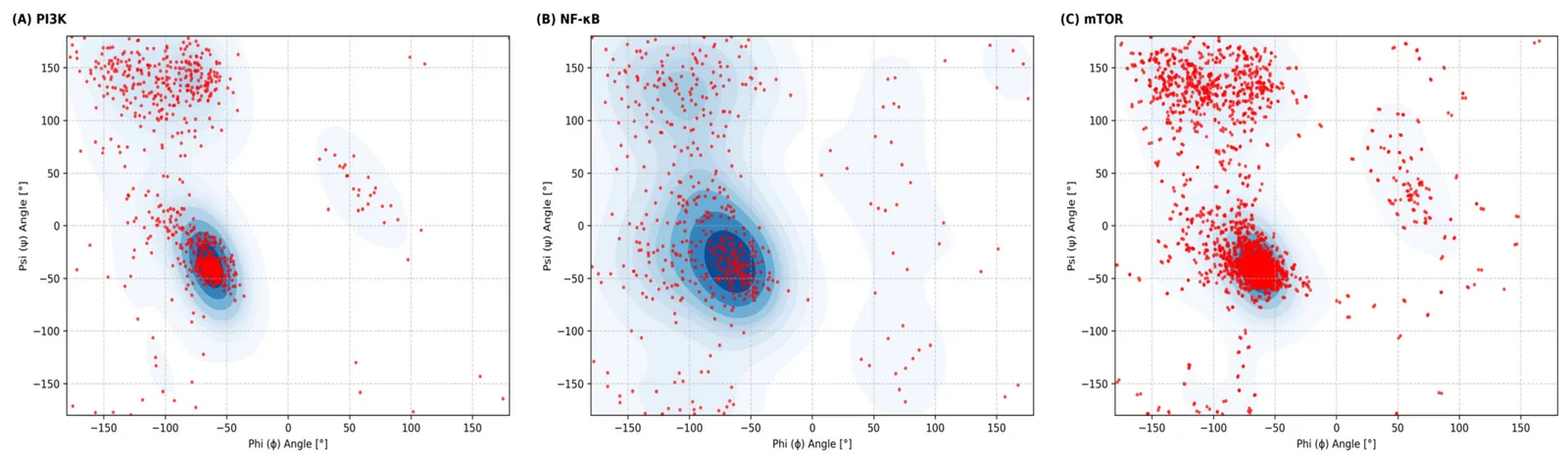
Ramachandran plots of the docked protein-ligand complexes: (**A**) PI3K, (**B**) NF-κB, and (**C**) mTOR. These plots are presented as a stereochemical quality assessment of the protein backbone. The color scale represents conformational regions, where dark blue indicates the most favored regions, light blue indicates additionally allowed regions, and white regions correspond to disallowed conformations. Red dots represent the φ (phi) and ψ (psi) dihedral angles of amino acid residues.

The Ramachandran plots (Figure 4) indicate that the protein structures predominantly occupy favored and additionally allowed conformational regions, consistent with the good stereochemical quality of the crystallographic models [60]. Because molecular docking was performed using rigid receptor structures, these plots primarily reflect the intrinsic backbone geometry of the experimentally determined protein structures rather than the accuracy of the predicted ligand binding poses. Therefore, the Ramachandran analysis is presented only as a general structural quality assessment and should not be considered a validation of the docking protocol. Validation of the docking methodology was instead indicated through native ligand redocking, which successfully reproduced the crystallographic binding geometries with best-pose RMSD values below 2.0 Å (Table 3).

#### 3.1.1. Validation of Molecular Docking by Native Ligand Redocking

**Figure 5:**
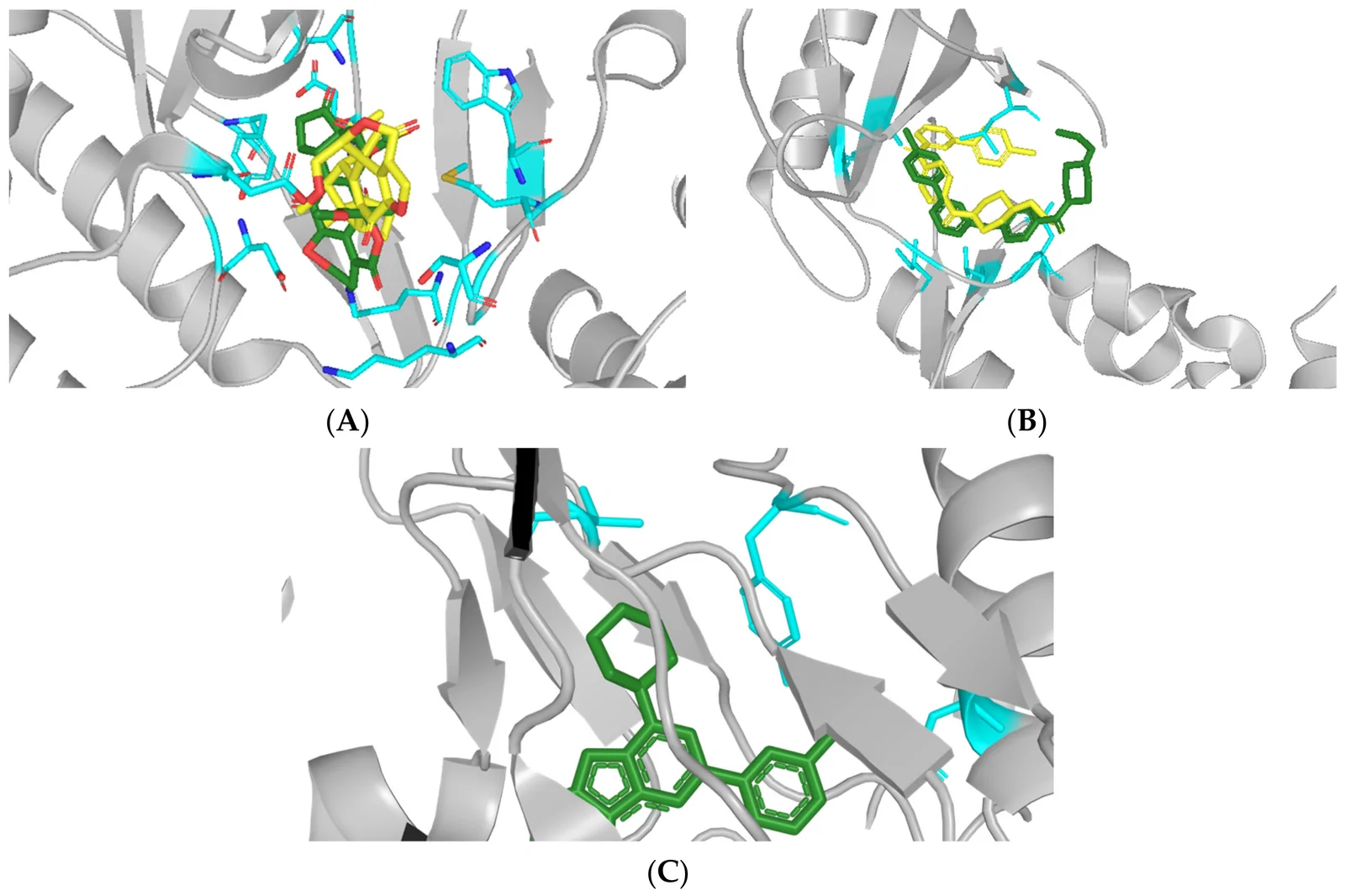
Native ligand redocking validation for (**A**) PI3K (PDB ID: 1E7U) with ligand KWT, (**B**) NF-κB (PDB ID: 3RZF) with ligand XNM, and (**C**) mTOR (PDB ID: 4JT6) with ligand X6K. In each panel, the crystallographic ligand is shown in green, the redocked pose in yellow, binding-site residues as cyan sticks, and the protein as a gray cartoon. The close overlap between the crystallographic and redocked conformations, with best-pose RMSD values of 0.239 Å (PI3K), 0.478 Å (NF-κB), and 0.162 Å (mTOR), confirms the accuracy and reliability of the docking protocol.

To validate the molecular docking protocol, the co-crystallized ligands KWT (PI3K, PDB ID: 1E7U), XNM (NF-κB, PDB ID: 3RZF), and X6K (mTOR, PDB ID: 4JT6) were extracted and redocked into their respective binding sites using the same AutoDock Vina protocol applied to the cinnamon-derived phytochemicals. The redocked poses closely reproduced the experimentally observed binding conformations, with best-pose RMSD values of 0.239 Å, 0.478 Å, and 0.162 Å for PI3K, NF-κB, and mTOR, respectively (Table 3). These values are well below the commonly accepted validation threshold of 2.0 Å, confirming that the docking protocol reliably reproduces the crystallographic binding geometries. The corresponding average RMSD values across all generated docking poses ranged from 2.428 to 2.881 Å, reflecting the expected conformational diversity among alternative docking solutions while maintaining accurate top-ranked poses. Comparison of the native ligand interactions with those of the cinnamon-derived phytochemicals showed that the phytochemicals occupied the same experimentally defined binding pockets and shared several key interacting residues with the native ligands. However, differences in the interaction networks were observed because the phytochemicals possess smaller molecular sizes and distinct chemical scaffolds, resulting primarily in hydrophobic and van der Waals interactions with fewer hydrogen bonds (Figure 5).

#### 3.1.2. Comparative Docking Analysis with Reference Inhibitors

To validate the docking results and benchmark the binding affinities of the cinnamon phytocompounds, known potential inhibitors of the target proteins were included for comparison. LY294002 or 2-(4-morpholinyl)-8-phenyl-4H-1-benzopyran-4-one (a standard PI3K inhibitor) [PubChem CID: 3973], BAY 11-7082 or (2E)-3-(4-methylbenzenesulfonyl) prop-2-enenitrile (NF-κB inhibitor) [PubChem CID: 5353431], and rapamycin (mTOR inhibitor) [PubChem CID: 5284616] were docked under identical computational parameters. The reference ligands exhibited binding energies of −8.1 kcal/mol (PI3K), −5.5 kcal/mol (NF-κB), and −5.6 kcal/mol (mTOR).

In contrast, the cinnamon-derived compounds displayed moderate binding affinities, with binding energies ranging from −4.9 to −6.5 kcal/mol toward target proteins. Although lower than those of potential synthetic inhibitors, the phytochemicals showed consistent interactions with key residues within each active site. Notably, p-cymene (−6.51 kcal/mol for mTOR) and cinnamic acid (−6.48 kcal/mol for mTOR) showed better binding energies among the tested compounds and were within the range reported for some low-molecular-weight allosteric modulators.

**Figure 6:**
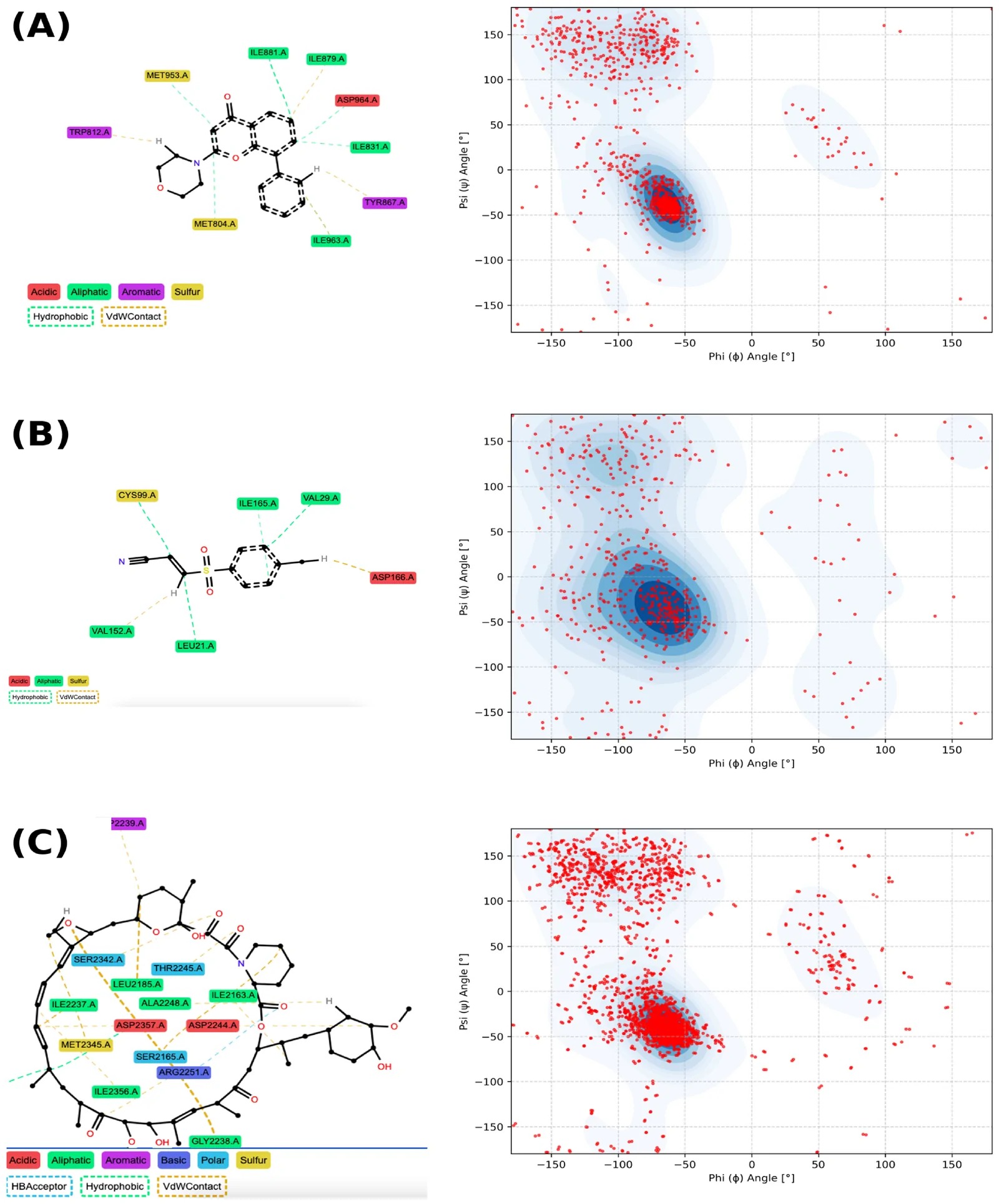
Two-dimensional interaction profiles and Ramachandran plot validation of protein reference ligand complexes: (**A**) PI3K–3973, (**B**) NF-κB–5353431, and (**C**) mTOR–5284616. Left panels show ligand–residue interactions, and right panels show Ramachandran plots. Interaction types are color-coded as acidic (red), aliphatic (green), aromatic (purple), sulfur (yellow), hydrophobic (light green), and van der Waals (orange dashed lines). Ramachandran color scale: dark blue (most favored), light blue (allowed), and white (disallowed); red dots indicate φ and ψ angles.

These findings suggest that natural compounds may serve as effective structural scaffolds for designing safer derivatives with improved potency, potentially acting as adjuvant modulators of the PI3K/AKT/mTOR and NF-κB pathways (Table 4, Figure 6).

#### 3.1.3. Comparison with Known Fragment Screening Studies

To contextualize our findings, we compared the binding energies of our cinnamon-derived compounds with those of indicated natural product fragments reported in the literature (Table 5). Quercetin, kaempferol, and genistein, well-studied flavonoids with molecular weights ranging from 270 to 302 Da, exhibit binding energies of −8.19 to −8.67 kcal/mol against PI3Kγ [61]. These compounds interact with key residues including Ile-831, Val-882, Met-953, and Ile-963, the same residues that stabilize our cinnamon compounds within the PI3K binding pocket, confirming overlapping binding sites despite lower absolute affinity.

Our compounds (p-cymene: 134 Da; eugenol: 164 Da; cinnamic acid: 148 Da) are significantly smaller fragments with fewer functional groups, naturally resulting in lower binding energies (−6.374 to −6.512 kcal/mol). This pattern is consistent with fragment-based drug discovery principles, where initial fragments (MW < 200 Da) typically show binding energies in the −5 to −7 kcal/mol range. For example, resveratrol (MW: 228 Da) shows binding energies of −6.6 to −6.8 kcal/mol against various cancer targets, demonstrating that the binding energy range of our compounds is consistent with bioactive natural product fragments [62].

**Table 5:** Comparison of binding energies between cinnamon-derived phytocompounds (this study) and indicated natural product fragments reported in the literature.

Ligand	Target Protein	AutoDock Vina (kcal/mol)	Molecular Weight (g/mol)	Reference
e-Cinnamaldehyde	mTOR	−6.330	132.16	This work
p-Cymene	mTOR	−6.512	134.22	This work
Eugenol	mTOR	−6.374	164.20	This work
Cinnamic Acid	mTOR	−6.488	148.16	This work
Quercetin	PI3Kγ	−8.19	302.23	Suhail et al., 2023 [61]
Kaempferol	PI3Kγ	−8.21	286.24	Suhail et al., 2023 [61]
Genistein	PI3Kγ	−8.67	270.24	Suhail et al., 2023 [61]
Resveratrol	PI3K-β	−5.98	228.25	Soliman et al., 2025 [63]
Curcumin	EGFR	−7.8	368.40	Shaik et al., 2019 [64]
Flavopiridol	PI3Kγ	−8.97	401.82	Suhail et al., 2023 [61]

#### 3.1.4. Consensus Molecular Docking Analysis of Cinnamon Phytochemicals

A consensus molecular docking strategy integrating AutoDock Vina, Smina, and GNINA was employed to evaluate the binding potential of four major cinnamon-derived phytochemicals, namely e-cinnamaldehyde, eugenol, p-cymene, and cinnamic acid, against three key therapeutic targets involved in colorectal cancer progression: phosphoinositide 3-kinase (PI3K; PDB ID: 1E7U), nuclear factor kappa B (NF-κB; PDB ID: 3RZF), and mammalian target of rapamycin (mTOR; PDB ID: 4JT6). The consensus docking results, including classical docking scores, GNINA-refined binding affinities, CNN pose scores, and CNN-predicted affinities, are summarized in Table 6.

For PI3K, all four phytochemicals exhibited favorable binding within the ATP-binding pocket, with Smina binding energies ranging from −5.60 to −6.10 kcal/mol. Eugenol and p-cymene produced the most favorable Smina binding energies (−6.10 kcal/mol), whereas cinnamic acid exhibited the most favorable GNINA-refined affinity (−5.92 kcal/mol). The CNN-based evaluation further indicated high-confidence binding poses for e-cinnamaldehyde (CNN pose score = 0.8992) and eugenol (CNN pose score = 0.8886). Among the investigated ligands, eugenol displayed the highest CNN-predicted affinity (4.928), suggesting a favorable interaction profile with the PI3K binding pocket. Collectively, these findings indicate that eugenol and cinnamic acid demonstrated the most favorable overall docking performance against PI3K when both classical and deep learning-based scoring metrics were considered.

Docking against NF-κB produced comparable binding energies for all phytochemicals, with Smina scores ranging from −5.20 to −5.70 kcal/mol. p-Cymene exhibited the lowest docking energies according to both Smina (−5.70 kcal/mol) and GNINA (−5.69 kcal/mol), whereas cinnamic acid achieved the highest CNN pose score (0.8186) and CNN-predicted affinity (4.914). The discrepancy between classical scoring functions and CNN-based rescoring suggests that different scoring algorithms emphasize distinct physicochemical features of ligand binding within the NF-κB active site. Consequently, p-cymene demonstrated the most favorable predicted binding energy, while cinnamic acid produced the highest-confidence binding geometry.

For mTOR, all four phytochemicals demonstrated favorable docking energies, with Smina binding energies ranging from −6.00 to −6.20 kcal/mol. Eugenol and cinnamic acid shared the lowest Smina binding energy (−6.20 kcal/mol), whereas cinnamic acid produced the most favorable GNINA affinity (−6.12 kcal/mol) and the highest CNN-predicted affinity (4.586). In contrast, e-cinnamaldehyde exhibited the highest CNN pose score (0.8799), indicating a highly plausible binding orientation within the kinase active site. The combined results suggest that cinnamic acid demonstrated the most consistent predicted interaction profile with mTOR across multiple scoring metrics.

Comparison of the docking results across the three molecular targets indicated differences in the predicted interaction profiles of the investigated phytochemicals. Eugenol showed comparatively lower predicted docking energies toward PI3K, p-cymene produced the lowest predicted docking energies for NF-κB, and cinnamic acid exhibited relatively consistent predicted docking scores across all three proteins, particularly for PI3K and mTOR.

Overall, the consensus docking analysis identified cinnamic acid as the most consistent multitarget candidate, while eugenol and p-cymene displayed greater target-specific binding preferences for PI3K and NF-κB, respectively. These computational predictions provide a rational basis for subsequent molecular dynamics simulations and MM/GBSA binding free-energy calculations to further investigate the stability and energetic favorability of the predicted protein–ligand complexes.

### 3.2. ADMET Analyses

The pharmacokinetic and toxicity profiles of e-cinnamaldehyde, eugenol, p-cymene, and cinnamic acid were evaluated using ADMETlab 3.0. The predicted physicochemical properties, drug-likeness parameters, pharmacokinetic characteristics, and toxicity endpoints are summarized in Table 7 and Table 8.

All four phytochemicals satisfied Lipinski’s rule of five and Veber’s criteria without any violations, indicating favorable physicochemical characteristics for oral drug development. The compounds exhibited relatively low molecular weights (132.06–164.08 g/mol), low topological polar surface areas (0.00–37.30 Å^2^), and acceptable lipophilicity (logP = 2.26–4.35). None of the compounds triggered PAINS alerts, suggesting a low likelihood of assay interference. Furthermore, all compounds showed a predicted bioavailability score of 0.55 and high gastrointestinal absorption, supporting their potential for oral administration.

Differences were observed in the predicted absorption and distribution characteristics. E-cinnamaldehyde was predicted to readily penetrate the blood–brain barrier (BBB), whereas eugenol and p-cymene exhibited partial BBB permeability, and cinnamic acid was predicted to have limited BBB penetration. Among the investigated compounds, p-cymene displayed the highest lipophilicity (logP = 4.35), which may favor membrane permeability but could also influence tissue distribution and plasma protein binding.

The metabolism predictions indicated compound-specific interactions with cytochrome P450 enzymes. Eugenol was predicted to inhibit both CYP3A4 and CYP2D6, whereas e-cinnamaldehyde was predicted to inhibit CYP2D6 only. In contrast, p-cymene and cinnamic acid showed no predicted inhibition of these two major CYP isoforms, suggesting a lower potential for CYP-mediated drug–drug interactions.

Predicted toxicity profiles revealed several differences among the phytochemicals. E-cinnamaldehyde was predicted to exhibit positive AMES mutagenicity, whereas eugenol showed a borderline prediction, and both p-cymene and cinnamic acid were predicted to be non-mutagenic. Possible hERG inhibition was predicted for e-cinnamaldehyde and eugenol, while p-cymene and cinnamic acid showed lower predicted hERG liability. Hepatotoxicity was predicted for all four compounds, with cinnamic acid exhibiting the highest predicted hepatotoxicity risk. However, all compounds were predicted to possess low acute toxicity based on estimated LD_50_ values and showed low predicted cytotoxicity.

**Figure 7:**
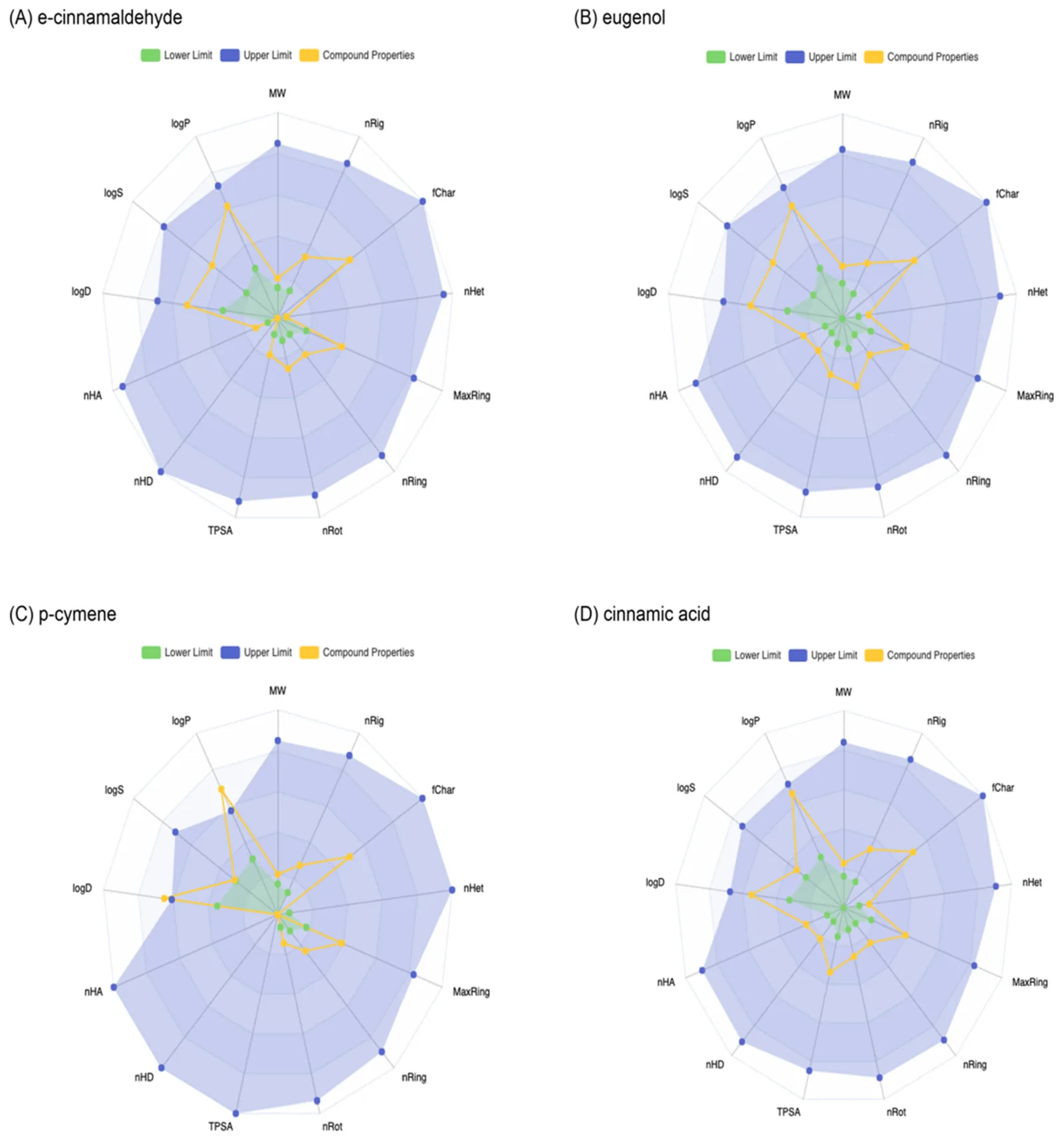
Radar plot summarizing the ADMET (Absorption, Distribution, Metabolism, Excretion, and Toxicity) analysis for (**A**) e-cinnamaldehyde, (**B**) eugenol, (**C**) p-cymene, and (**D**) cinnamic acid, comparing their properties to indicate lower and upper limits for drug-like compounds, where green represents the lower limit, blue represents the upper limit, and orange represents the compound properties.

The radar plot analysis (Figure 7) demonstrated that the overall physicochemical properties of all four phytochemicals remained within the recommended ranges for drug-like molecules. Nevertheless, the predicted metabolic liabilities and toxicity endpoints differed among the compounds, indicating that although the investigated phytochemicals possess favorable drug-likeness and absorption characteristics, their safety profiles may require further experimental validation. Overall, eugenol exhibited the most balanced combination of oral drug-likeness and pharmacokinetic properties, whereas p-cymene displayed favorable metabolic characteristics with minimal CYP inhibition, and cinnamic acid demonstrated desirable physicochemical properties despite a higher predicted hepatotoxicity risk.

### 3.3. Molecular Dynamics Simulation Studies

Although molecular docking is an essential method in drug design, it lacks high accuracy. Therefore, a more precise computational technique, such as molecular dynamics simulation (MDs), is needed to complement docking studies. By using Newton’s equations of motion to determine the velocity and position of each atom inside the protein-ligand complex during the course of the simulation, MD simulations typically offer thorough insight into molecular behavior [65]. This technique captures various essential biomolecular activities, including protein folding, conformational changes, and ligand binding, at the femtosecond timescale. The trajectories generated from the MD simulations present a three-dimensional movie that depicts the positioning of the system atoms at each point during the simulation [66].

Thus, MD simulations can provide additional insights into the dynamic behavior of ligand-protein complexes beyond docking, although their interpretability depends on simulation time and system setup.

After consensus docking and ADMET evaluation, molecular dynamics (MD) simulations were performed to investigate the dynamic stability of the selected protein-ligand complex under physiological conditions. Based on the initial AutoDock Vina screening, the mTOR–p-cymene complex exhibited the most favorable docking energy (−6.512 kcal/mol) and was therefore selected for further MD analysis. The MD simulation was conducted on the initially selected complex to evaluate its structural stability during prolonged simulation.

The simulation was carried out for 100 ns, and the trajectories were analyzed using root mean square deviation (RMSD), root mean square fluctuation (RMSF), radius of gyration (Rg), hydrogen bonding, and additional structural descriptors to characterize the stability of the protein-ligand complex.

#### 3.3.1. Root Mean Square Deviation (RMSD) Analysis

The root mean square deviation (RMSD) is a parameter used to assess the mean deviation of conformational changes in protein-ligand complexes at a particular time. In general, RMSD provides valuable information about the extent of divergence exhibited by a set of atoms (such as a protein, ligand, or ligand-protein complex) from the original reference structure, enabling an evaluation of the system’s stability [67]. High fluctuations in the RMSD values indicate instability within the complex, likely resulting from conformational or orientation changes that occur during the simulation. The irregular patterns observed in the RMSD values indicate unsuitable accommodation of the ligands within the binding pockets over the MD simulation timeframes [68].

**Figure 8:**
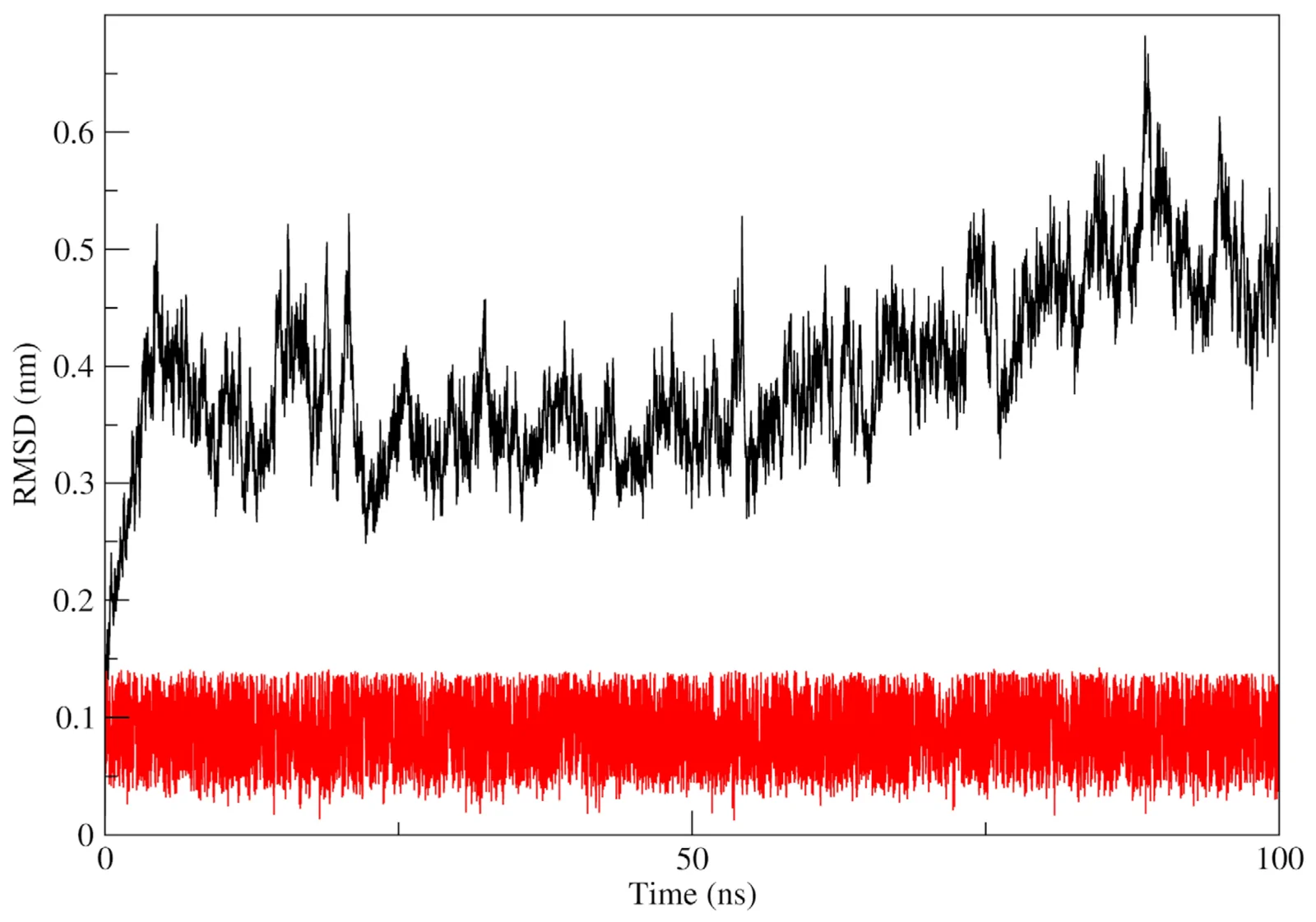
Root mean square deviation (RMSD) profiles of the mTOR–p-cymene complex during the 100 ns molecular dynamics simulation. The black curve represents the RMSD of the protein backbone, while the red curve corresponds to the ligand RMSD relative to the initial docked structure. The protein exhibited rapid equilibration followed by moderate conformational fluctuations, whereas the ligand maintained consistently low RMSD values throughout the simulation, indicating stable retention within the mTOR binding pocket.

The conformational stability of the mTOR–p-cymene complex was evaluated by monitoring the RMSD of the protein backbone and ligand throughout the 100 ns simulation (Figure 8). The protein backbone RMSD increased rapidly during the first 5–10 ns, reaching approximately 0.35–0.40 nm, which is consistent with structural equilibration after system relaxation. Following this initial adjustment, the RMSD fluctuated between 0.30 and 0.45 nm for most of the simulation, indicating that the protein maintained an overall stable conformation. During the final 20–30 ns, a gradual increase in RMSD was observed, with transient peaks approaching 0.65 nm, suggesting enhanced flexibility of surface loops or local conformational rearrangements rather than global structural destabilization.

In contrast, the ligand RMSD remained consistently low throughout the simulation, fluctuating between approximately 0.04 and 0.13 nm. The absence of large ligand deviations indicates that p-cymene remained stably positioned within the mTOR binding pocket despite moderate conformational changes in the protein backbone.

Overall, the RMSD analysis demonstrates that the protein-ligand complex remained structurally stable throughout the 100 ns simulation, with ligand binding preserved despite moderate backbone flexibility.

#### 3.3.2. Root Mean Square Fluctuation (RMSF) Analysis

Residue-level flexibility of the mTOR–p-cymene complex was evaluated using root mean square fluctuation (RMSF) analysis over the 100 ns molecular dynamics simulation (Figure 8). The RMSF profile showed that most residues exhibited relatively low fluctuations, with values predominantly ranging between 0.13 and 0.25 nm, indicating that the overall protein architecture remained structurally stable throughout the simulation. The average flexibility remained below the threshold of 0.35 nm for the majority of residues, suggesting limited backbone mobility and preservation of the folded conformation.

**Figure 9:**
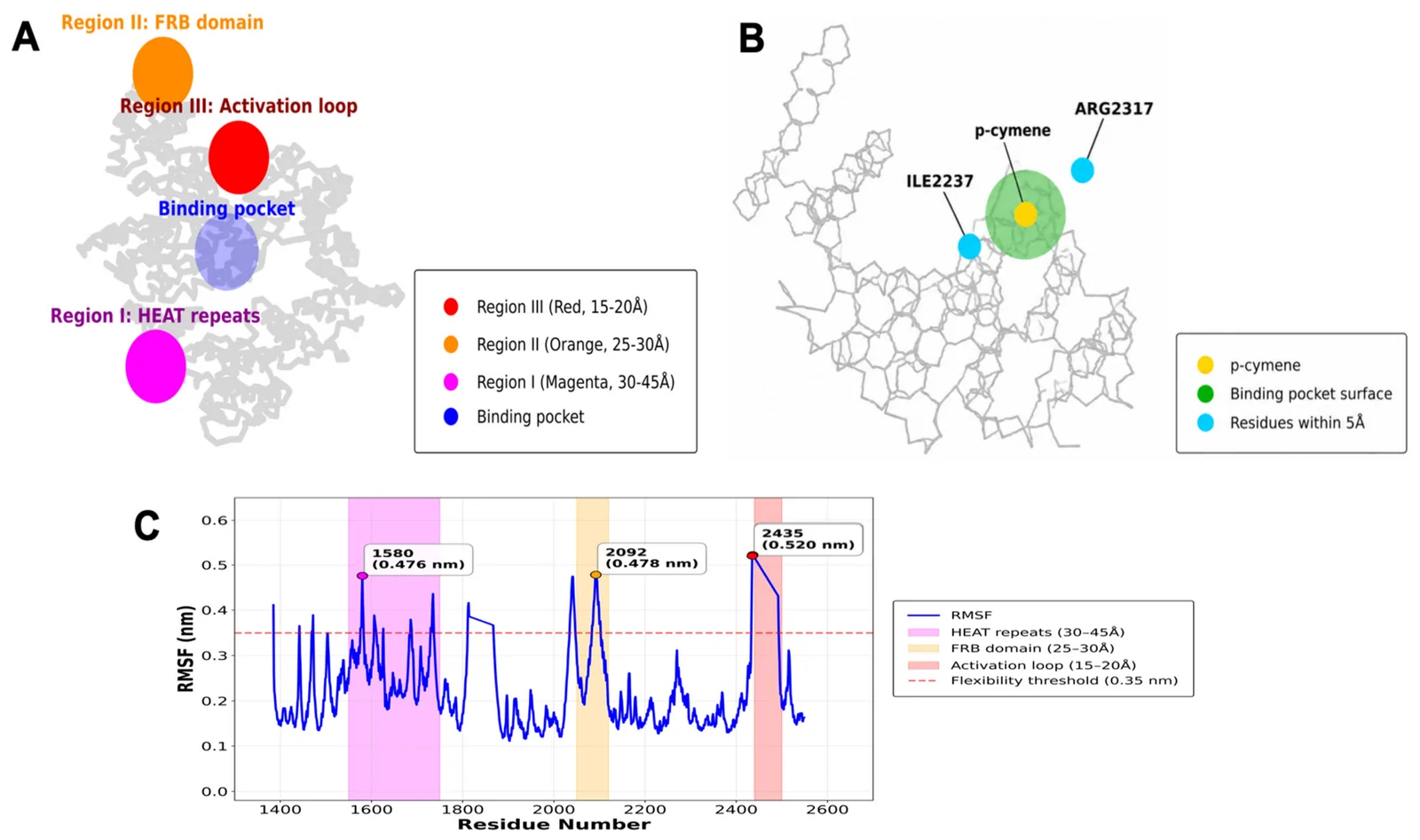
Structural mapping of RMSF during the 100 ns molecular dynamics simulation of the mTOR–p-cymene complex. (**A**) Major RMSF regions mapped onto the three-dimensional structure of mTOR. The activation-loop region (red), FRB-associated region (orange), and HEAT-repeat region (magenta) represent the principal flexibility hotspots identified during the simulation, whereas the ligand-binding pocket is shown in blue. (**B**) Enlarged view of the p-cymene binding pocket. The ligand is shown as yellow sticks, the binding pocket surface is shown in green, and residues within 5 Å of the ligand are highlighted. (**C**) RMSF profile of Cα atoms during the 100 ns simulation. The principal fluctuation peaks correspond to the structural regions shown in Panel A. The dashed red line indicates the flexibility threshold (0.35 nm). The colored background regions in Panel C (magenta, orange, and red) correspond directly to the structural domains color-coded in Panel A using the same color scheme.

Three localized regions displayed comparatively higher flexibility (Figure 9C). The first prominent peak was observed within the HEAT-repeat region (approximately residues 1550–1750), reaching a maximum RMSF of 0.476 nm around residue 1580. A second region of elevated flexibility occurred in the FRB-associated region (approximately residues 2035–2045), where the RMSF reached approximately 0.457 nm. The largest fluctuation (0.520 nm) was detected within the activation-loop region (approximately residues 2435–2450), indicating that this regulatory segment undergoes greater conformational motion than the structured core of the protein.

To provide structural context for these fluctuations, the major RMSF peaks were mapped onto the three-dimensional structure of mTOR (Figure 9A,B). The structural mapping revealed that the highly flexible regions are predominantly associated with peripheral structural and regulatory elements, including the HEAT-repeat and activation-loop regions, whereas residues forming the immediate p-cymene binding pocket (within 5 Å of the ligand) exhibited comparatively low fluctuations. This observation suggests that the ligand-binding site remained conformationally stable during the simulation, while the higher RMSF values primarily reflect the intrinsic flexibility of solvent-exposed loops and regulatory domains rather than local instability of the binding pocket.

The preservation of a relatively rigid ligand-binding environment, together with the localized nature of the observed fluctuations, indicates that the flexibility is unlikely to compromise ligand binding. Instead, these motions most likely represent the inherent dynamic behavior of regulatory regions of mTOR that are known to undergo conformational adjustments during protein function. Consistent with the stable backbone and ligand RMSD profiles, the RMSF analysis further supports the overall structural stability of the mTOR–p-cymene complex throughout the 100 ns molecular dynamics simulation.

#### 3.3.3. Radius of Gyration (Rg) Analysis

**Figure 10:**
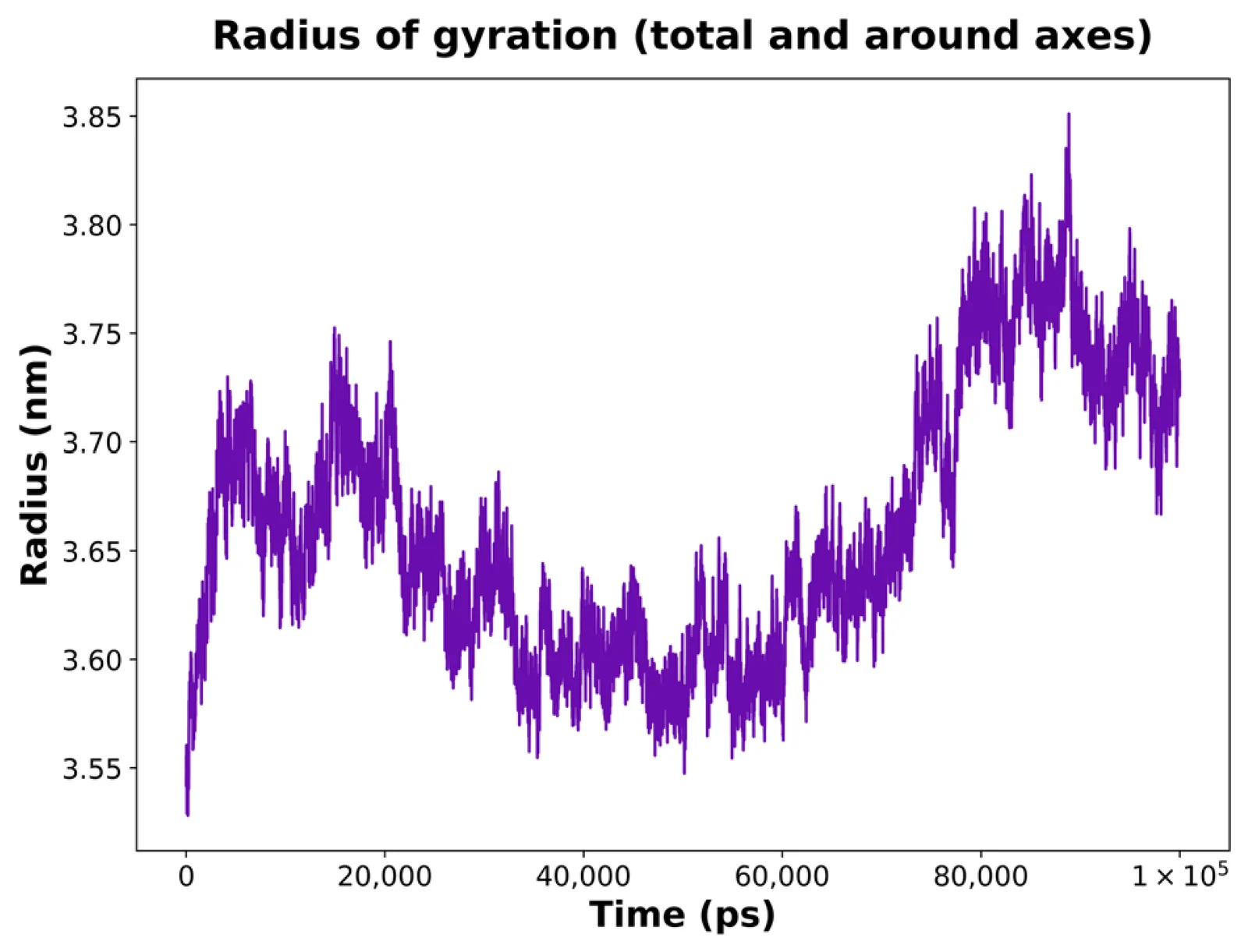
Radius of gyration (Rg) profile of the mTOR–p-cymene complex during the 100 ns molecular dynamics simulation. The Rg values remained within a narrow range (3.55–3.85 nm), indicating preservation of the overall compactness of the protein. A modest increase in Rg observed during the later stage of the simulation is consistent with normal conformational flexibility rather than structural destabilization.

The radius of gyration (Rg) was calculated to evaluate the overall compactness of the mTOR–p-cymene complex during the 100 ns molecular dynamics simulation (Figure 10). Throughout the simulation, the Rg values remained within a relatively narrow range of 3.55–3.85 nm, indicating that the global structural organization of the protein was maintained without evidence of large-scale unfolding or structural collapse.

During the initial phase of the simulation (0–10 ns), the Rg increased from approximately 3.55 nm to 3.70 nm, reflecting structural relaxation following system equilibration. Subsequently, the complex maintained relatively stable Rg values between 3.58 and 3.63 nm for the majority of the simulation (approximately 20–70 ns), suggesting preservation of the protein’s compact conformation. A gradual increase in Rg was observed after approximately 75 ns, with values reaching 3.75–3.80 nm before stabilizing toward the end of the trajectory. This modest increase likely reflects normal conformational breathing motions rather than substantial structural expansion.

Overall, the limited variation in the Rg profile demonstrates that the mTOR–p-cymene complex retained its global compactness throughout the 100 ns simulation. When considered together with the RMSD and RMSF analyses, these results indicate that the protein maintained its structural integrity while accommodating moderate conformational flexibility during ligand binding.

#### 3.3.4. Solvent Accessible Surface Area (SASA) Analysis

**Figure 11:**
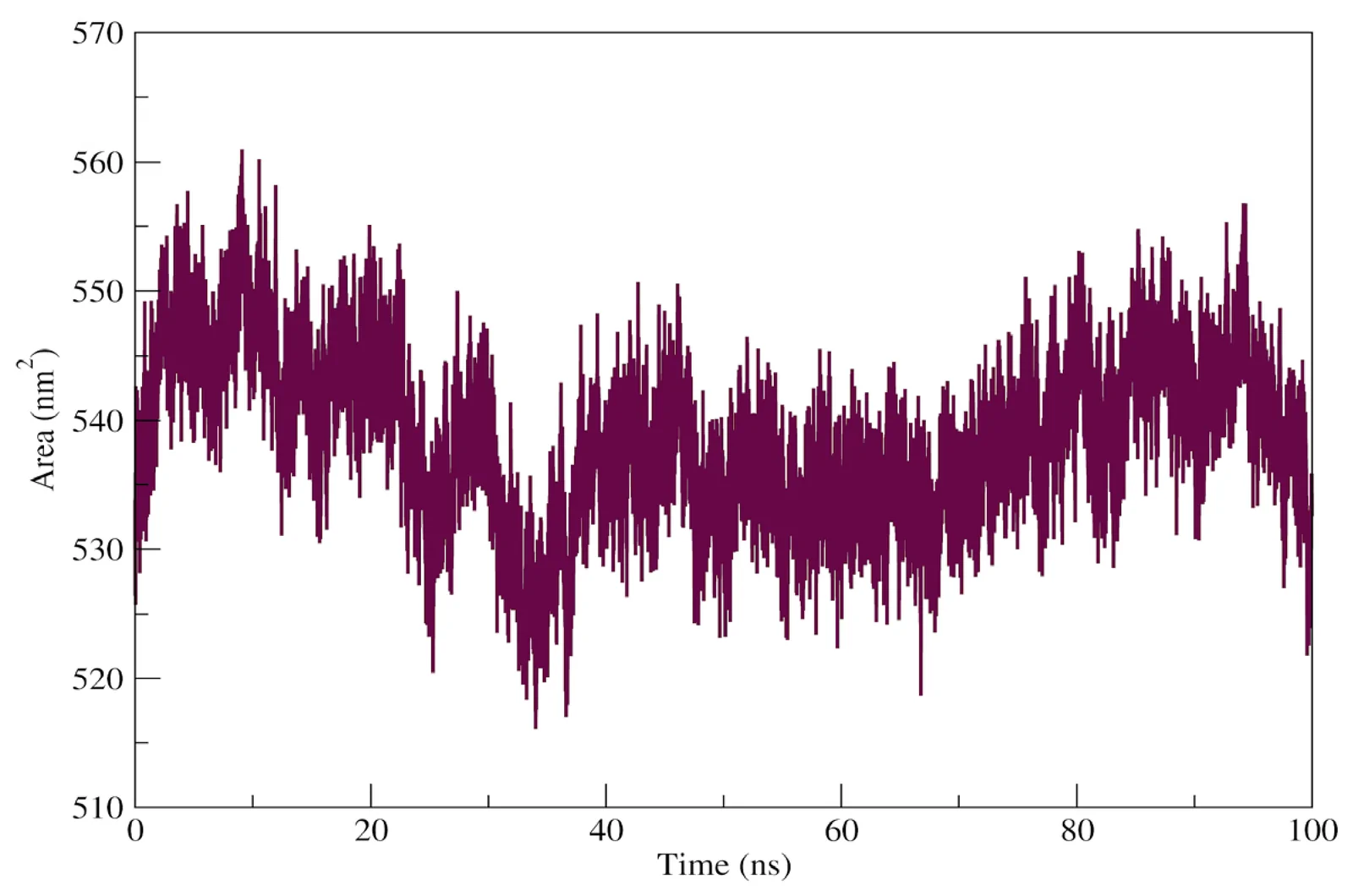
Solvent accessible surface area (SASA) profile of the mTOR–p-cymene complex during the 100 ns molecular dynamics simulation. The SASA values remained within a relatively narrow range (516–561 nm^2^), indicating stable solvent exposure of the protein surface. Moderate fluctuations observed during the simulation are consistent with normal conformational dynamics rather than major structural rearrangements.

The solvent accessible surface area (SASA) was calculated to evaluate changes in solvent exposure and overall protein surface accessibility during the 100 ns molecular dynamics simulation (Figure 11). Throughout the simulation, SASA values fluctuated between approximately 516 and 561 nm^2^, with most of the trajectory remaining within the range of 530–550 nm^2^, indicating relatively stable solvent exposure of the protein surface.

During the initial 10–15 ns, the complex exhibited comparatively higher SASA values (approximately 545–555 nm^2^), reflecting structural relaxation following system equilibration. A moderate decrease in SASA was observed between approximately 30 and 40 ns, reaching values of 520–530 nm^2^, which is consistent with a transient increase in structural compactness. Subsequently, the SASA gradually increased and stabilized between 540 and 550 nm^2^ during the final stage of the simulation, suggesting restoration of solvent exposure following normal conformational fluctuations.

Overall, the SASA profile exhibited only moderate variations throughout the simulation, with no evidence of sustained increases or decreases indicative of major structural rearrangements. These observations are consistent with the RMSD, RMSF, and radius of gyration analyses, collectively indicating that the mTOR–p-cymene complex maintained its overall structural integrity while undergoing normal dynamic fluctuations under simulated physiological conditions.

#### 3.3.5. Hydrogen Bond Analysis

**Figure 12:**
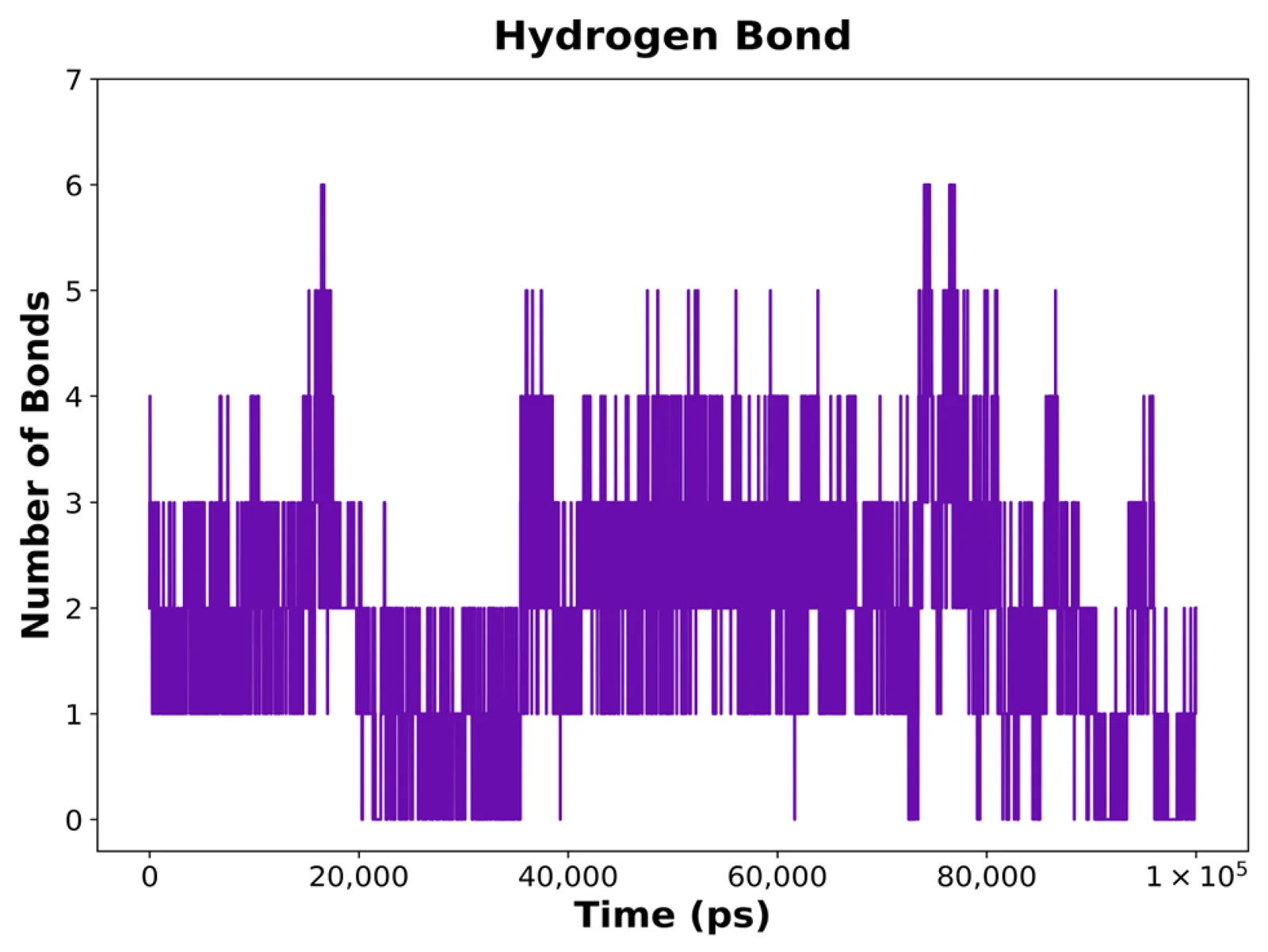
Evolution of the hydrogen-bond network during the 100 ns molecular dynamics simulation of the mTOR–p-cymene system. The reported hydrogen-bond counts represent the overall hydrogen-bonding network within the simulated system and should not be interpreted as direct hydrogen bonds between p-cymene and mTOR, as p-cymene lacks hydrogen-bond donor and acceptor atoms.

The hydrogen-bond network of the simulated mTOR–p-cymene system was monitored throughout the 100 ns molecular dynamics simulation to evaluate the overall stability of hydrogen-bonding interactions within the system (Figure 12). The total number of hydrogen bonds exhibited dynamic fluctuations during the simulation, reflecting the natural conformational flexibility of the protein and the surrounding solvent environment. Variations in hydrogen-bond counts over time indicate continuous rearrangement of the hydrogen-bonding network while maintaining the structural integrity of the simulated system.

It is important to note that p-cymene is an aromatic hydrocarbon lacking hydrogen-bond donor and acceptor atoms and, therefore, is not expected to form direct hydrogen bonds with mTOR. Accordingly, the hydrogen-bond analysis was not interpreted as representing direct protein–ligand interactions but rather as describing changes in the overall hydrogen-bonding environment during the simulation. The observed fluctuations are primarily attributable to the dynamic reorganization of intramolecular protein hydrogen bonds and protein–solvent hydrogen-bond interactions accompanying conformational motions of the protein.

The molecular docking and molecular dynamics analyses were collectively consistent with the maintenance of the predicted mTOR–p-cymene binding pose throughout the 100 ns simulation. The low ligand RMSD, stable protein backbone RMSD, limited fluctuations within the binding pocket observed in the RMSF analysis, relatively stable radius of gyration, consistent solvent-accessible surface area, and MM/GBSA binding free-energy estimates together suggest that p-cymene remained associated with the predicted hydrophobic binding pocket during the simulation. These computational observations further suggest that the predicted ligand–protein interaction was predominantly mediated by hydrophobic and van der Waals interactions rather than direct hydrogen bonding.

#### 3.3.6. MM/GBSA Binding Free Energy Analysis

**Figure 13:**
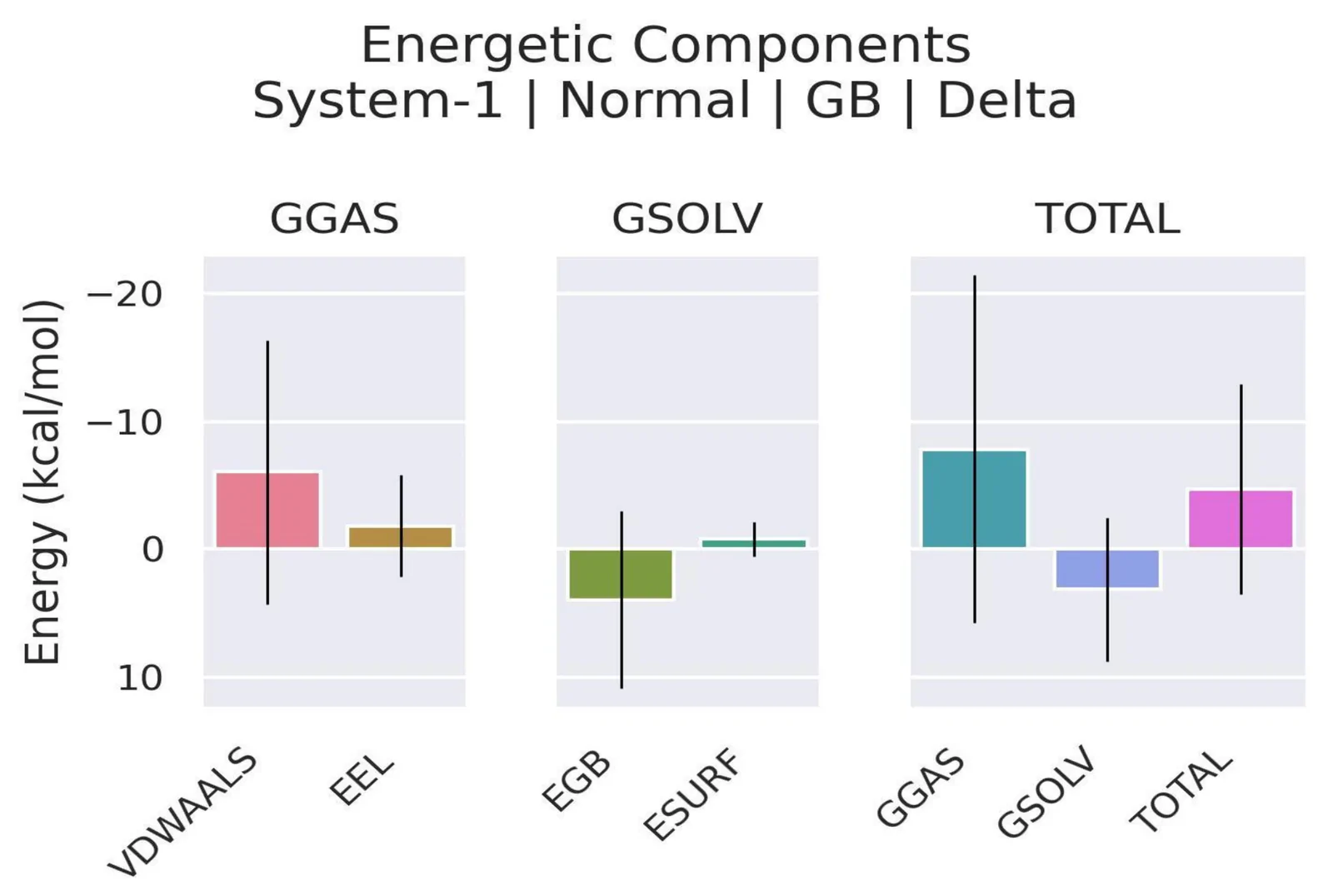
MM/GBSA binding free-energy decomposition for the mTOR–p-cymene complex following the 100 ns molecular dynamics simulation. The energetic contributions of van der Waals (VDWAALS), electrostatic (EEL), polar solvation (EGB), and non-polar solvation (ESURF) interactions are shown together with the total binding free energy. The predominance of favorable van der Waals interactions indicates that hydrophobic contacts are the major driving force stabilizing the protein-ligand complex.

To further characterize the energetic profile of the mTOR–p-cymene complex, the binding free energy was estimated using the Molecular Mechanics/Generalized Born Surface Area (MM/GBSA) method. The energy decomposition results are summarized in Table 9 and illustrated in Figure 13.

The MM/GBSA calculations yielded an overall binding free energy (ΔG_bind) of −4.70 ± 8.20 kcal/mol, indicating a modest binding interaction between p-cymene and mTOR under the simulated conditions. The van der Waals interaction (ΔE_vdW = −6.03 ± 10.33 kcal/mol) represented the largest favorable energetic contribution, suggesting that hydrophobic contacts were the principal contributors to ligand binding. Electrostatic interactions also contributed favorably (ΔE_ele = −1.82 ± 3.94 kcal/mol), although their contribution was smaller than that of van der Waals interactions.

The polar solvation energy (ΔG_GB = +3.94 ± 6.88 kcal/mol) opposed complex formation, as commonly observed in aqueous environments, whereas the non-polar solvation component (ΔG_SA = −0.79 ± 1.35 kcal/mol) contributed favorably. Consequently, the favorable gas-phase interaction energy (ΔG_gas = −7.85 ± 13.60 kcal/mol) was partially offset by the unfavorable solvation energy (ΔG_solv = +3.15 ± 5.59 kcal/mol), resulting in an overall negative binding free energy.

The predominance of van der Waals interactions is consistent with the hydrophobic nature of p-cymene and agrees with the interaction patterns observed in the molecular docking analysis and throughout the molecular dynamics simulation. However, the calculated ΔG_bind indicates a relatively modest binding strength and should therefore be interpreted as a comparative estimate of ligand affinity rather than evidence of a high-affinity interaction. Taken together with the molecular dynamics results, the MM/GBSA analysis suggests that the mTOR–p-cymene complex remained structurally stable during the simulation despite exhibiting only modest predicted binding free energy.

### 3.4. Preliminary In Vitro Evaluation of Biological Potential of Cinnamon Extract

Although these assays do not directly confirm anticancer activity, they provide initial experimental support for the biological potential of cinnamon-derived compounds.

#### 3.4.1. H_2_O_2_ Reducing Ability

**Figure 14:**
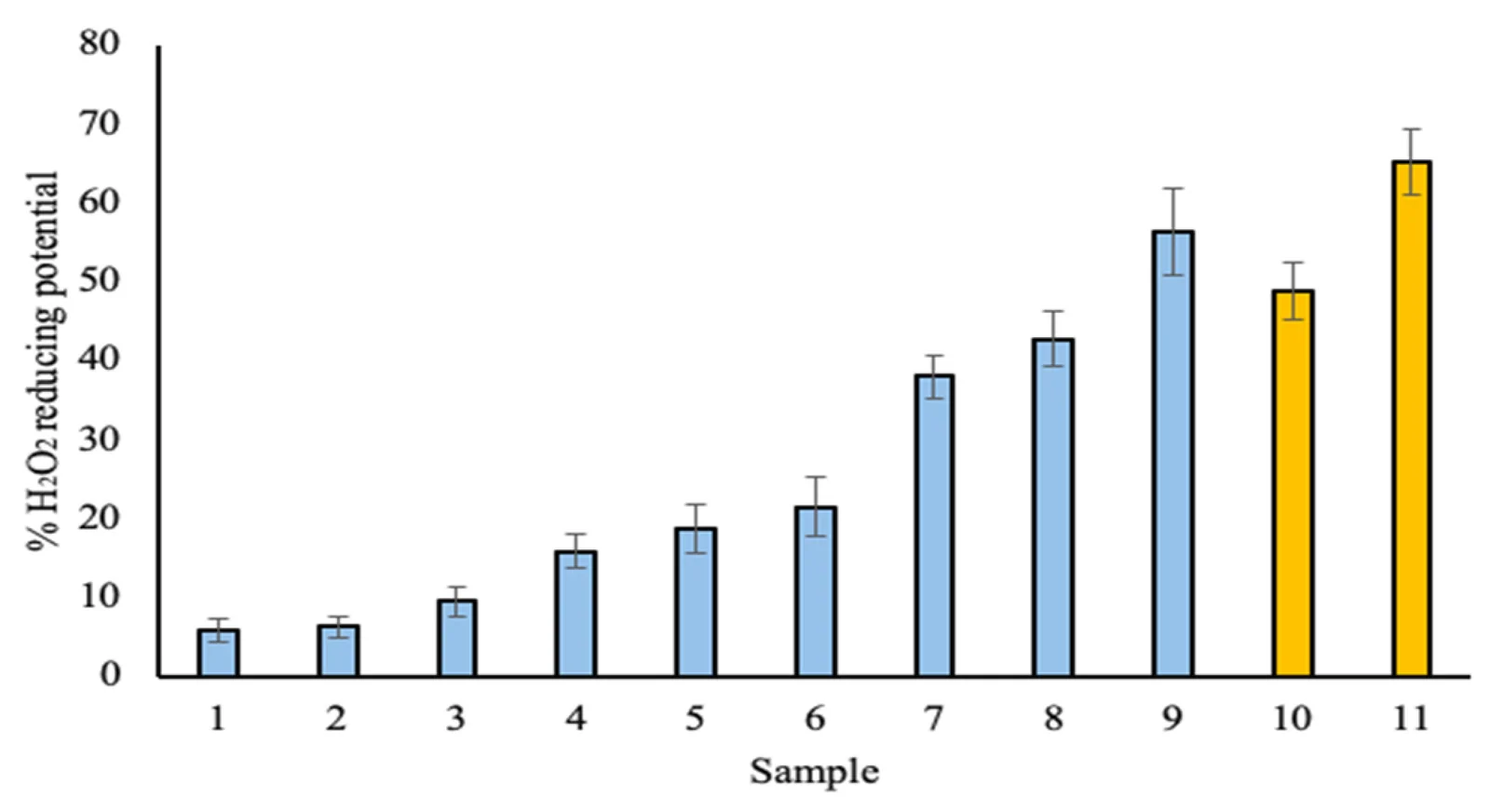
Hydrogen peroxide (H_2_O_2_) scavenging activity of the extract at different concentrations. Blue bars (1–9) represent extract concentrations of 50, 75, 100, 150, 200, 300, 400, 500, and 600 µg/mL, while orange bars (10 and 11) represent ascorbic acid at 100 and 200 µg/mL, respectively. Data are presented as mean ± SEM (*n* = 3, *p* < 0.05).

The cinnamon extract showed a noticeable ability to scavenge hydrogen peroxide, and this effect increased gradually with increasing extract concentration (Figure 14). Since hydrogen peroxide can generate highly reactive hydroxyl radicals, its neutralization is important in reducing oxidative stress. The antioxidant activity observed may be related to the presence of polyphenolic compounds in the extract, which can donate electrons and stabilize free radicals. These findings suggest that cinnamon possesses considerable antioxidant potential.

#### 3.4.2. DPPH Free Radical Scavenging Activity

**Figure 15:**
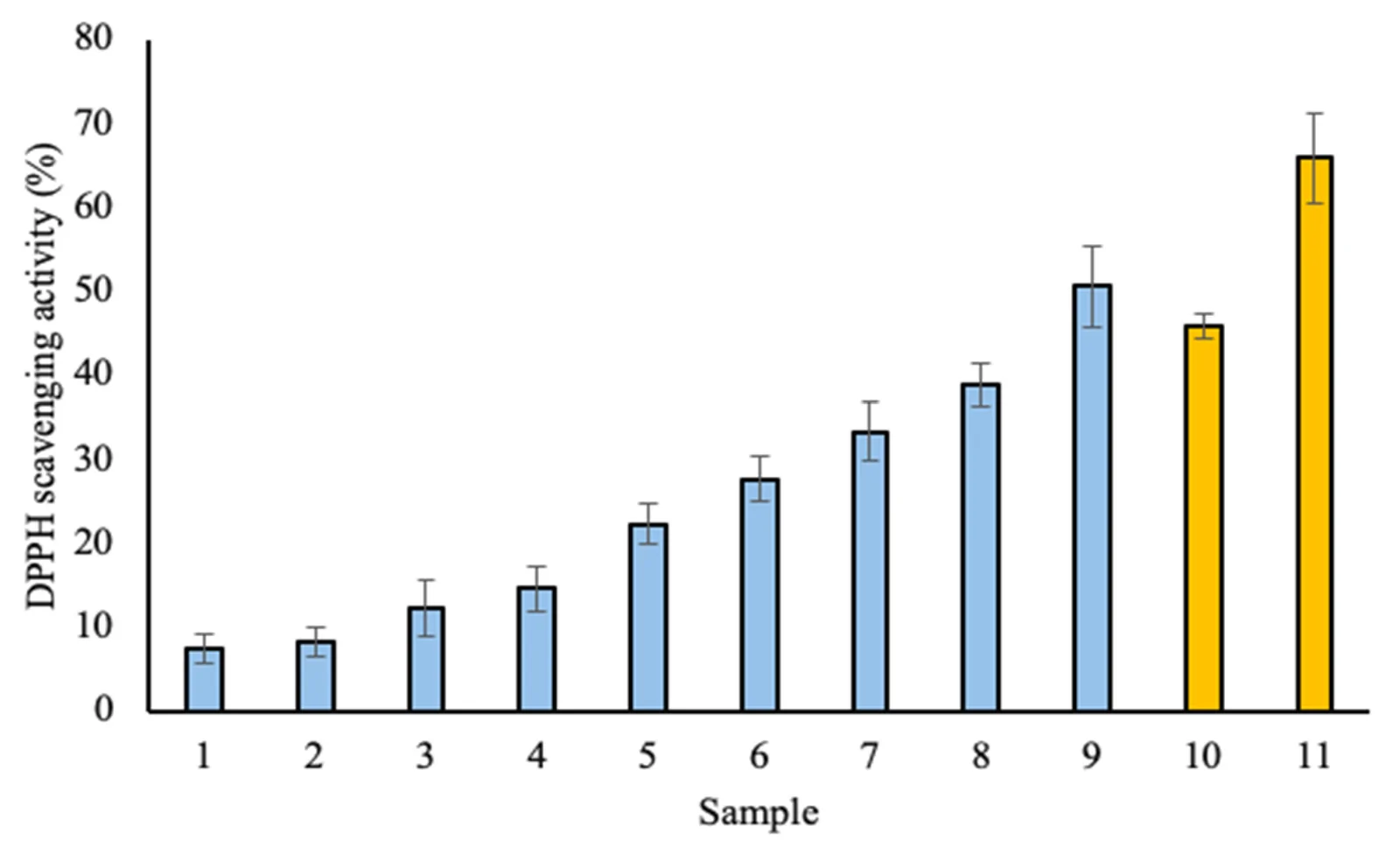
DPPH radical scavenging activity (%). Blue bars (1–9) represent extract concentrations of 50, 75, 100, 150, 200, 300, 400, 500, and 600 µg/mL, while orange bars (10 and 11) represent ascorbic acid at 100 and 200 µg/mL, respectively. Data are expressed as mean ± SEM (*n* = 3, *p* < 0.05).

The DPPH assay demonstrated that cinnamon extract effectively scavenged free radicals in a concentration-dependent manner (*p* < 0.05) (Figure 15). Higher extract concentrations resulted in greater DPPH inhibition, indicating strong antioxidant activity. The results were comparable to the standard antioxidant, ascorbic acid.

#### 3.4.3. Albumin Denaturation Inhibition

**Figure 16:**
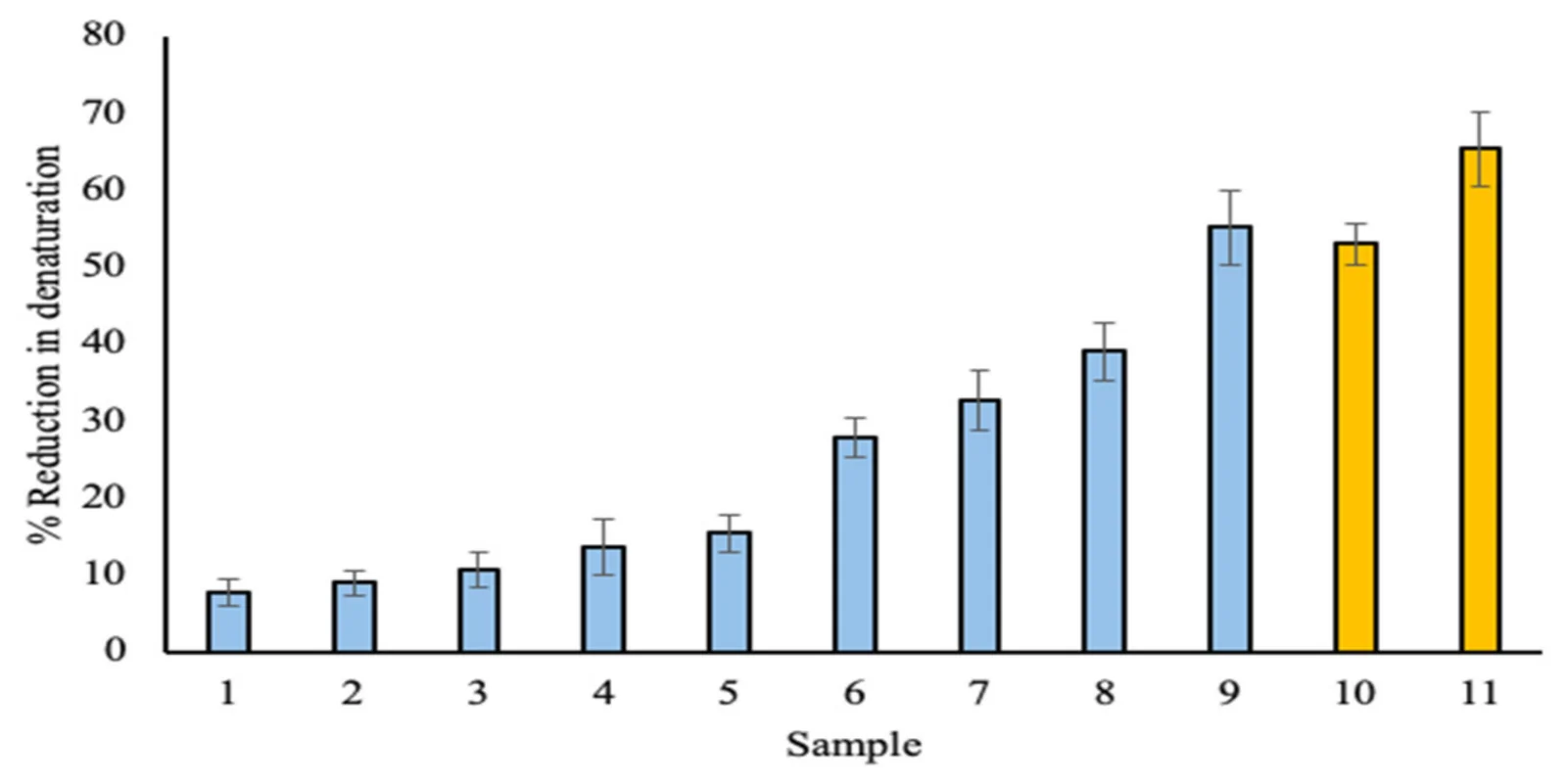
Percentage inhibition of albumin denaturation by extract. Blue bars (1–9) represent different concentrations of cinnamon extract (50, 75, 100, 150, 200, 300, 400, 500, and 600 µg/mL), while orange bars (10 and 11) indicate ibuprofen at concentrations of 100 and 200 µg/mL, respectively. Data are presented as mean ± SEM (*n* = 3, *p* < 0.05).

The anti-inflammatory activity of cinnamon extract was evaluated through inhibition of heat-induced albumin denaturation. The extract showed progressive protection against protein denaturation with increasing concentrations (*p* < 0.05) (Figure 16). Ibuprofen exhibited stronger inhibition. Although lower than the standard drug, the results indicate that cinnamon extract possesses measurable anti-inflammatory activity.

### 3.5. Cytotoxic and Antiproliferative Effects of Cinnamon Extract on Colorectal Cancer Cells

**Figure 17:**
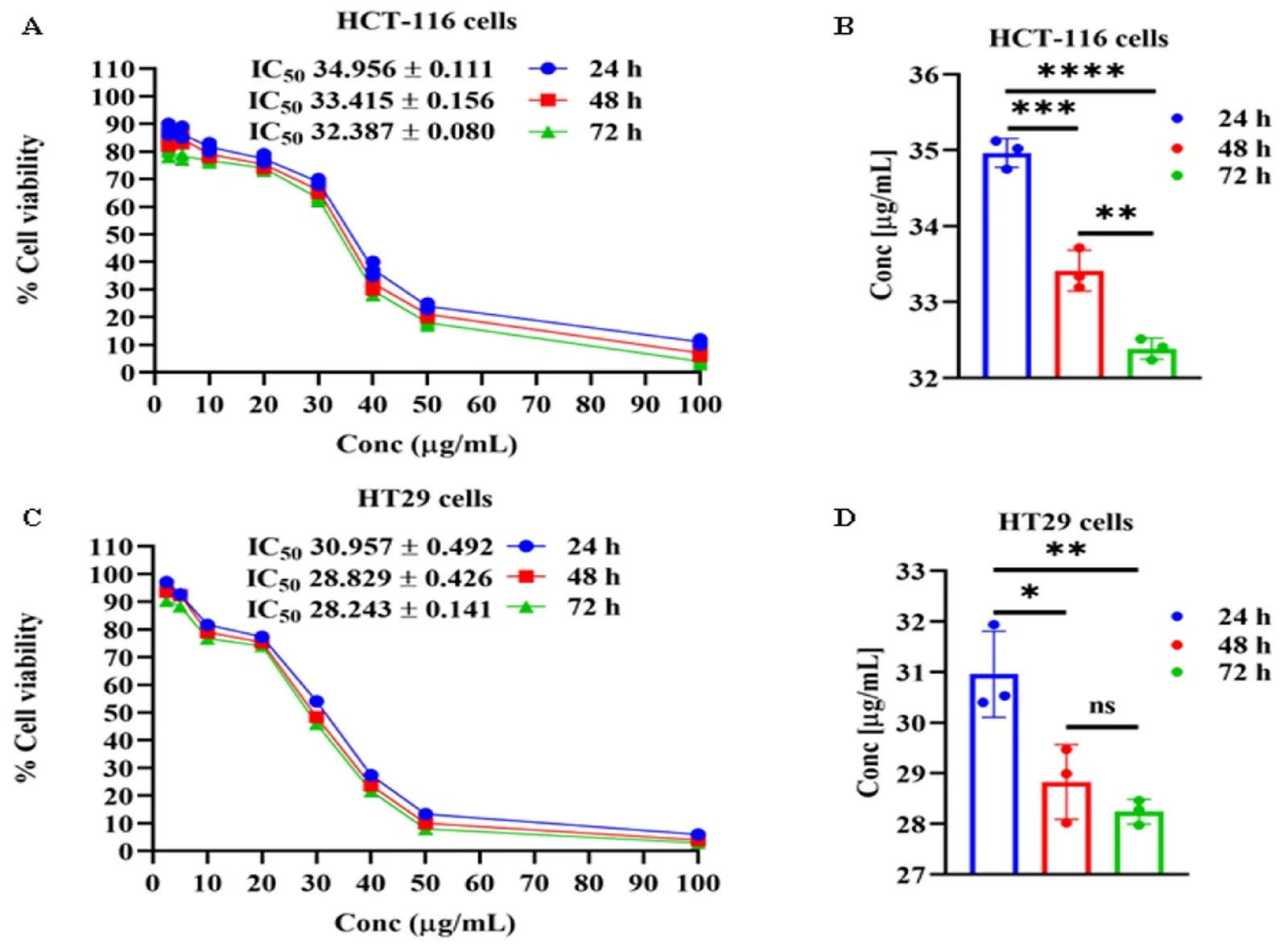
The methanolic extract of cinnamon reduced cell viability and proliferation in HCT-116 and HT29 colorectal cancer cell lines. (**A**) HCT-116 cells exhibited a dose-dependent decrease in viability at 24, 48, and 72 h, as measured by cell viability assays. IC_50_ values for each time point are shown in the graph. (**B**) Viable HCT-116 cell counts decreased significantly with longer treatment. (**C**) HT29 cells also showed reduced viability at all time points, with IC_50_ values presented in the graph. (**D**) Viable HT29 cell counts declined with prolonged exposure, indicating reduced proliferation. Data are presented as mean ± SEM from three experiments. Statistical significance was determined using one-way ANOVA and post hoc tests. Significance is indicated as *p* < 0.05 (*), *p* < 0.01 (**), *p* < 0.001 (***), *p* < 0.0001 (****); ns denotes not significant.

The methanolic extract of cinnamon demonstrated strong, dose and time dependent cytotoxic and antiproliferative effects on HCT-116 and HT29 colorectal cancer cell lines. In HCT-116 cells (Figure 17A), higher extract concentrations reduced viability at 24, 48, and 72 h, with IC_50_ values decreasing over time, indicating increased cytotoxicity. HT29 cells (Figure 17C) also showed reduced viability at all concentrations and time points, with lower IC_50_ values after prolonged treatment. Cell counts suggested these findings: HCT-116 cells (Figure 17B) had fewer viable cells at 48 and 72 h than at 24 h, and HT29 cells (Figure 17D) showed reduced viability after treatment. These results indicate that the extract effectively suppresses colorectal cancer cell growth and viability.

## 4. Discussion

The present study integrated consensus molecular docking, ADMET prediction, molecular dynamics (MD) simulation, MM/GBSA binding free-energy analysis, and preliminary in vitro validation to investigate the multitarget anticancer potential of major cinnamon-derived phytochemicals against three key proteins involved in colorectal cancer progression, namely PI3K, NF-κB, and mTOR. Unlike many previous computational investigations that focused on a single protein target or individual phytochemicals, this work employed a comprehensive workflow to evaluate the interactions of e-cinnamaldehyde, eugenol, p-cymene, and cinnamic acid across multiple interconnected oncogenic signaling pathways. Such a multitarget strategy is particularly relevant because dysregulation of the PI3K/AKT/mTOR and NF-κB signaling pathways frequently occurs simultaneously during colorectal cancer development and contributes to uncontrolled proliferation, inflammation, angiogenesis, metastasis, apoptosis evasion, and therapeutic resistance [69,70].

The consensus docking analysis integrating AutoDock Vina, Smina, and GNINA provided complementary evidence for ligand binding while reducing dependence on a single scoring function. Classical docking and deep learning-based rescoring identified target-specific binding preferences among the investigated phytochemicals. Eugenol demonstrated the most favorable overall interaction profile with PI3K, exhibiting strong Smina and GNINA binding affinities together with the highest CNN-predicted affinity. In contrast, p-cymene produced the most favorable classical docking energies against NF-κB, whereas cinnamic acid achieved the highest CNN confidence scores and emerged as the most consistent multitarget ligand across PI3K, NF-κB, and mTOR. The integration of empirical scoring functions with convolutional neural network-based rescoring increases confidence in the predicted binding modes by combining complementary approaches for evaluating protein-ligand interactions [71,72,73,74].

The observed docking scores are consistent with previous reports demonstrating that natural phytochemicals can effectively interact with cancer-associated signaling proteins. Larger flavonoids, including quercetin, kaempferol, genistein, and flavopiridol, generally exhibit stronger docking energies because of their larger molecular size and greater capacity to establish hydrogen-bonding and electrostatic interactions with kinase active sites [61]. Likewise, curcumin has been reported to interact favorably with EGFR and other oncogenic signaling proteins through multiple hydrogen-bonding interactions [64].

Nevertheless, docking scores obtained from independent studies should not be directly compared because they depend on receptor structures, docking algorithms, search-space definitions, and scoring functions. Despite their comparatively small molecular weights (132–164 Da), the cinnamon-derived phytochemicals investigated in this study exhibited predicted docking scores within the range commonly reported for fragment-sized natural compounds. These computational observations suggest that the investigated phytochemicals may serve as fragment-sized starting points for further optimization, although their biological relevance requires experimental validation.

To further assess the docking protocol, the predicted binding modes of the native co-crystallized ligands were compared with those of the selected cinnamon-derived phytochemicals. The native ligands and phytochemicals were predicted to occupy the same experimentally defined binding pockets and shared several interacting residues, indicating that the phytochemicals were docked within the crystallographically characterized active sites. As expected, differences in the interaction networks were observed because the phytochemicals possess smaller molecular sizes and distinct chemical scaffolds, resulting in fewer predicted hydrogen-bonding interactions and a greater contribution from hydrophobic and van der Waals contacts. In addition, native ligand redocking reproduced the experimentally observed binding orientations for PI3K, NF-κB, and mTOR with best-pose RMSD values below 2.0 Å, indicating that the docking protocol was able to reproduce the crystallographic binding geometries under the applied docking conditions.

The predicted pharmacokinetic properties further supported the therapeutic potential of the investigated compounds. All four phytochemicals satisfied Lipinski’s rule of five and Veber’s criteria without PAINS alerts, indicating favorable oral drug-likeness. High predicted gastrointestinal absorption together with acceptable physicochemical properties suggests that these compounds possess suitable characteristics for oral administration. However, predicted CYP450 inhibition, hepatotoxicity, and possible hERG liabilities indicate that additional experimental pharmacokinetic and toxicological evaluation will be necessary before clinical translation. Among the investigated compounds, eugenol demonstrated the most balanced overall ADMET profile, whereas p-cymene exhibited minimal CYP inhibition despite its comparatively higher lipophilicity.

To evaluate whether the predicted docked complex remained stable under dynamic conditions, the mTOR–p-cymene complex was selected for molecular dynamics simulation based on the initial AutoDock Vina screening, in which p-cymene exhibited the most favorable docking energy toward mTOR. Although the subsequent consensus docking analysis identified cinnamic acid as the most consistent multitarget ligand, the MD simulation independently demonstrated that the initially selected complex remained structurally stable throughout the 100 ns simulation. RMSD, RMSF, radius of gyration, solvent-accessible surface area, and hydrogen-bond analyses consistently indicated preservation of the overall protein architecture while allowing moderate conformational flexibility characteristic of biologically active proteins [66,75].

The MM/GBSA-derived binding free energy of −4.70 kcal/mol suggests that p-cymene exhibits a modest interaction with the mTOR binding site. This observation is consistent with the small size of p-cymene, which provides limited opportunities for hydrogen bonding and electrostatic interactions compared with larger kinase inhibitors. Nevertheless, the MD simulation demonstrated that the ligand remained stably accommodated within the binding pocket throughout the simulation period. Therefore, p-cymene should be regarded as a preliminary scaffold or fragment suitable for future optimization rather than a potent mTOR inhibitor. Moreover, because MM/GBSA calculations neglect configurational entropy and depend on the selected force field and implicit solvent model, the calculated binding free energy should be interpreted as a comparative rather than an absolute measure of ligand affinity.

The computational analyses were complemented by preliminary experimental evaluation of crude methanolic cinnamon extract, which demonstrated antioxidant activity and concentration-dependent cytotoxic effects in HCT-116 and HT-29 cancer cell lines. However, because these experiments were performed using a crude extract rather than isolated phytochemicals, the observed biological effects cannot be directly attributed to the four compounds evaluated in the computational study. Instead, these findings provide preliminary evidence of the overall anticancer potential of cinnamon extract and warrant further investigation using purified phytochemicals to validate their individual biological activities and mechanisms of action.

Despite these encouraging findings, several limitations should be acknowledged. Molecular docking, ADMET prediction, and molecular dynamics simulations provide computational estimates rather than direct measurements of biological activity. One limitation of the present study is that molecular dynamics simulation and MM/GBSA analysis were performed only for the mTOR-p-cymene complex. Although consensus docking identified cinnamic acid as the most consistent multitarget ligand and eugenol exhibited favorable interactions with PI3K, detailed dynamic characterization of these additional complexes was beyond the scope of the present work because of computational resource constraints. Future studies will extend molecular dynamics simulations and free-energy analyses to additional high-performing protein-ligand complexes to enable a more comprehensive comparison of their dynamic behavior and binding energetics. The biological validation was performed using crude cinnamon extract rather than purified phytochemicals. Future investigations should therefore include enzyme inhibition assays, apoptosis and cell-cycle analyses, transcriptomic profiling, and in vivo studies to validate the predicted molecular mechanisms and therapeutic potential of the individual phytochemicals.

Overall, the integration of consensus molecular docking, ADMET prediction, molecular dynamics simulation, MM/GBSA binding free-energy analysis, and preliminary experimental validation provides a comprehensive assessment of the anticancer potential of cinnamon-derived phytochemicals. Among the investigated compounds, eugenol demonstrated the strongest interaction profile toward PI3K, p-cymene exhibited favorable dynamic stability with mTOR, and cinnamic acid emerged as the most consistent multitarget candidate across the investigated signaling proteins. Collectively, these findings provide a rationale for further preclinical evaluation of cinnamon-derived phytochemicals as prospective multitarget anticancer agents.

Although molecular docking, molecular dynamics simulations, and MM/GBSA analyses provide valuable insights into potential binding modes and relative binding affinities, these computational approaches cannot demonstrate direct enzymatic inhibition or modulation of intracellular signaling pathways. Therefore, the present findings should be regarded as hypothesis-generating and require validation through biochemical enzyme inhibition assays and pathway-specific cellular experiments.

## 5. Conclusions

This study presents an integrated computational and preliminary experimental evaluation of major cinnamon-derived phytochemicals targeting the PI3K/NF-κB/mTOR signaling network. Consensus molecular docking using AutoDock Vina, Smina, and GNINA predicted that e-cinnamaldehyde, eugenol, p-cymene, and cinnamic acid are capable of interacting with the selected cancer-associated proteins. Among the evaluated compounds, eugenol exhibited comparatively lower predicted docking energies toward PI3K, p-cymene was selected for molecular dynamics simulation based on its predicted docking profile with mTOR, and cinnamic acid showed relatively consistent predicted docking scores across PI3K, NF-κB, and mTOR. Collectively, these computational analyses illustrate how multiple docking and rescoring approaches can be combined to compare predicted ligand-protein interaction profiles.

In silico ADMET analysis predicted that all four phytochemicals satisfy common drug-likeness criteria and possess predicted oral bioavailability. However, the predicted CYP450 interactions and potential hepatotoxicity indicate that additional pharmacological and toxicological investigations are required before drawing conclusions regarding their therapeutic potential.

The molecular dynamics simulation was consistent with maintenance of the predicted mTOR-p-cymene binding pose throughout the 100 ns simulation. MM/GBSA analysis yielded a modest binding free-energy estimate (ΔG_bind = −4.70 ± 8.20 kcal/mol), accompanied by considerable energetic variability. Therefore, these computational results should be interpreted cautiously as qualitative predictions of the simulated protein-ligand interaction rather than definitive evidence of binding affinity or target inhibition. Additional replicate simulations, longer simulation times, and complementary free-energy calculations would further strengthen the computational assessment.

Preliminary in vitro experiments showed that a crude methanolic cinnamon bark extract reduced the viability of HCT-116 and HT-29 colorectal cancer cells in a concentration- and time-dependent manner. Because these experiments were performed using a crude extract rather than isolated phytochemicals, they cannot be directly attributed to the specific compounds evaluated computationally, nor do they establish inhibition of the PI3K, NF-κB, or mTOR signaling pathways. Instead, these preliminary biological observations are consistent with the reported biological activity of cinnamon extracts and provide a rationale for further investigation of their individual bioactive constituents.

Overall, the computational analyses provide predictions of potential protein-ligand interactions that may assist in prioritizing cinnamon-derived phytochemicals for future investigation. Together with the preliminary observations obtained using the crude cinnamon extract, these findings provide a basis for subsequent studies involving purified compounds, biochemical enzyme inhibition assays, pathway-specific cellular analyses, advanced biophysical characterization, expanded molecular dynamics simulations, and in vivo validation to determine whether the predicted interactions have biological and therapeutic relevance.

## Figures and Tables

**Table 1:** Docking interactions of cinnamon phytochemicals with target proteins.

Ligand (PubChem CID)	Target Protein	Binding Affinity (kcal/mol)	Key Interacting Residues	Interaction Type(s)
e-Cinnamaldehyde (637511)	PI3K	−5.261	Ile831, Ile879, Ile881, Ile963, Met953, Val882, Tyr867	Hydrophobic
	NF-κB	−4.919	Val29, Leu21	Hydrophobic
	mTOR	−6.330	Arg2316, Leu2303, Tyr2320	H-bond, Hydrophobic
Eugenol (3314)	PI3K	−5.629	Ile831, Ile879, Ile963, Val882, Met804, Glu88, Tyr867, Met953, Trp812	H-bond, Hydrophobic
	NF-κB	−5.240	Val74, Val29, Ala42, Ile165, Leu21, Met96	Hydrophobic
	mTOR	−6.374	Tyr2225, Ile2356, Met2345	H-bond, Hydrophobic
p-Cymene (7463)	PI3K	−6.103	Ile831, Ile879, Ile881, Ile963, Met804, Met953, Val882, Lys833, Tyr867	Hydrophobic, π-alkyl
	NF-κB	−5.684	Glu97, Met96, Val29, Leu21, Ile165, Ala42, Val74	H-bond, Hydrophobic
	mTOR	−6.512	Trp2313, Arg2348, Ile2237, Met2345, Arg2317, Trp2239, Ile2356, Tyr2225, Ile2303	H-bond, Hydrophobic, π-π stacking
Cinnamic acid (444539)	PI3K	−5.370	Ile831, Ile879, Ile963, Tyr867,Val882, Met953	H-bond, Hydrophobic
	NF-κB	−5.050	Leu21, Val29, Tyr98	H-bond, Hydrophobic
	mTOR	−6.488	Tyr2225, Ile2303, Ile2356, Met2345, Trp2239	H-bond, Hydrophobic, π-π stacking

**Table 2:** Docking grid parameters used for molecular docking of target proteins (PI3K, NF-κB, and mTOR), including grid center coordinates and box dimensions defined for AutoDock Vina simulations.

Protein	Enzyme Class (EC)	Grid Center Coordinates (Å)	Grid Box Dimensions (Å)
PI3K	2.7.1.153	23.45, 62.15, 20.62	13.18, 12.42, 13.04
NF-κB	2.7.11.10	90.97, −23.19, 54.21	9.59, 18.90, 12.89
mTOR	2.7.11.1	16.88, −16.68, −51.88	85.48, 42.05, 22.88

**Table 3:** Native ligand redocking validation and key interacting residues of PI3K, NF-κB, and mTOR.

Protein Target	PDB ID	Native Ligand	Binding Affinity (kcal/mol)	Best RMSD (Å)	Average RMSD (Å)	Key Interacting Residues (Distance, Å)	Interaction Type(s)
PI3K	1E7U	KWT (Kinase Inhibitor)	−8.40	0.239	2.428	ILE963 (4.24), ASP964 (5.07), MET804 (5.08), ILE831 (5.45), ILE879 (5.55), LYS833 (5.65), MET953 (5.88), PRO810 (6.27)	H-bond, Hydrophobic, π-alkyl
NF-κB	3RZF	XNM (IκB Kinase Inhibitor)	−8.30	0.478	2.881	LEU21 (3.32), ASP103 (4.41), TYR98 (4.51)	H-bond, Hydrophobic
mTOR	4JT6	X6K (mTOR Kinase Inhibitor)	−8.10	0.162	2.430	LEU2185 (3.54), ILE2356 (3.65), ILE2237 (4.63), MET2345 (4.82)	Hydrophobic, π-alkyl

**Table 4:** Docking results of reference inhibitors against target proteins. Reference ligands were docked under the same grid parameters used for cinnamon phytochemicals.

Protein (PDB Id)	Reference Ligand (PubChem CID)	Grid Center Coordinates (Å)	Grid Box Dimensions (Å)	Binding Affinity (kcal/mol)
PI3K (1E7U)	3973	23.45, 62.15, 20.62	13.18, 12.42, 13.04	−8.1
NF-κB (3RZF)	5353431	90.97, −23.19, 54.21	9.59,18.90, 12.89	−5.5
mTOR (4JT6)	5284616	16.88, −16.68, −51.88	85.48, 42.05, 22.88	−5.673

**Table 6:** Consensus molecular docking scores of cinnamon phytochemicals against PI3K, NF-κB, and mTOR.

Ligand	Target Protein	AutoDock Vina (kcal/mol)	Smina (kcal/mol)	GNINA (kcal/mol)	Intramolecular Energy (kcal/mol)	CNN Pose Score	CNN Predicted Affinity
e-Cinnamaldehyde	PI3K	−5.261	−5.60	−5.59	−0.12	0.8992	4.281
	NF-κB	−4.919	−5.20	−5.14	−0.12	0.7911	4.241
	mTOR	−6.330	−6.00	−5.81	−0.12	0.8799	4.415
Eugenol	PI3K	−5.629	−6.10	−5.93	−0.17	0.8886	4.928
	NF-κB	−5.240	−5.50	−5.16	−0.25	0.7103	4.545
	mTOR	−6.374	−6.20	−5.59	−0.24	0.6951	4.212
p-Cymene	PI3K	−6.103	−6.10	−5.09	−0.16	0.7540	4.208
	NF-κB	−5.684	−5.70	−5.69	−0.17	0.5658	3.996
	mTOR	−6.512	−6.00	−5.83	−0.17	0.7628	4.259
Cinnamic acid	PI3K	−5.370	−5.90	−5.92	−0.21	0.8693	4.620
	NF-κB	−5.050	−5.50	−5.33	−0.20	0.8186	4.914
	mTOR	−6.488	−6.20	−6.12	−0.16	0.7989	4.586

**Table 7:** Simplified Molecular Input Line Entry System (SMILES) notation of the selected cinnamon-derived phytochemicals used for docking and ADMET analysis.

S. N.	Ligand	SMILES
1	e-Cinnamaldehyde	C1=CC=C(C=C1)/C=C/C=O
2	Eugenol	COC1=C(C=CC(=C1)CC=C)O
3	p-Cymene	CC1=CC=C(C=C1)C(C)C
4	Cinnamic acid	C1=CC=C(C=C1)/C=C/C(=O)O

**Table 8:** Summary of drug-likeness and ADMETlab 3.0 predictions for e-cinnamaldehyde, eugenol, p-cymene, and cinnamic acid. Properties include physicochemical descriptors, oral drug-likeness rules, ADMET indicators, and in silico toxicity risks. These values were used to assess the oral bioavailability and safety profiles of each compound.

Parameter	e-Cinnamaldehyde	Eugenol	p-Cymene	Cinnamic Acid
Molecular weight (g/mol)	132.06	164.08	134.11	148.05
H-bond donors	0	1	0	1
H-bond acceptors	1	2	0	2
LogP	2.26	2.32	4.35	2.62
Rotatable bonds	2	3	1	2
TPSA (Å^2^)	17.07	29.46	0.00	37.30
Lipinski violations	0	0	0	0
Veber violations	0	0	0	0
PAINS alert	None	None	None	None
Bioavailability score	0.55	0.55	0.55	0.55
BBB penetration	Yes	Partial	Partial	No
GI absorption	High	High	High	High
Synthetic accessibility (0 easy–10 hard)	2.14	2.62	2.01	2.35
CYP3A4 inhibitor	No	Yes	No	No
CYP2D6 inhibitor	Yes	Yes	No	No
hERG inhibition	Possible	Possible	Low	Low
AMES mutagenicity	Positive	Borderline	Negative	Negative
Carcinogenicity	Possible	Possible	Low	Low
Hepatotoxicity	Yes	Yes	Yes	High
Cytotoxicity	No	Low	Low	Low
Predicted LD50 (mg/kg, rat)	Low toxicity	Low toxicity	Low toxicity	Low toxicity

**Table 9:** MM/GBSA binding free-energy decomposition of the mTOR–p-cymene complex following the 100 ns molecular dynamics simulation.

Energy Component	Mean (kcal/mol)	SD (kcal/mol)	SEM (kcal/mol)	Contribution to Binding
ΔEvdW (VDWAALS)	−6.03	10.33	0.23	Favorable
ΔEele (EEL)	−1.82	3.94	0.09	Favorable
ΔGGB (Polar solvation)	+3.94	6.88	0.15	Unfavorable
ΔGSA (Non-polar solvation)	−0.79	1.35	0.03	Favorable
ΔGgas (GGAS)	−7.85	13.60	0.30	Favorable
ΔGsolv (GSOLV)	+3.15	5.59	0.12	Unfavorable
ΔGbind (TOTAL)	−4.70	8.20	0.18	Modest binding interaction

## Data Availability

The code supporting this study is available on GitHub (https://github.com/Ravindra-Raut/cinnamon) (accessed on 28 June 2026).

## Abbreviations

The following abbreviations are used in this manuscript:

ADMET	Absorption, Distribution, Metabolism, Excretion, and Toxicity
AKT	Protein kinase B
CADD	Computer-aided drug design
COX	Cyclooxygenase
MD	Molecular dynamics
mTOR	Mammalian target of rapamycin
NF-κB	Nuclear factor kappa B
PCA	Principal component analysis
PI3K	Phosphatidylinositol-3-kinase
QSAR	Quantitative structure-activity relationship
Rg	Radius of gyration
RMSD	Root mean square deviation
RMSF	Root mean square fluctuation
SAR	Structure-activity relationship
SMILES	Simplified molecular input line entry system
STAT3	Signal transducer and activator of transcription 3
VEGF	Vascular endothelial growth factor
